# Coherent-resonant netting: disorder-enhanced selectivity from transient wave-like dynamics on biological connectomes

**DOI:** 10.3389/fncom.2026.1813959

**Published:** 2026-05-13

**Authors:** Oleg Dolgikh

**Affiliations:** Independent Researcher, Sant Cugat del Vallès, Spain

**Keywords:** connectome transport, decision architecture, dephasing, disorder-enhanced selectivity, mechanistically neutral model, structural graph

## Abstract

Biological agents face an energy-information bottleneck: inference requires rapid exploration of large hypothesis spaces, yet high-gain spiking is metabolically expensive. We propose Coherent-Resonant Netting (CRN) as a two-regime decision architecture in which a low-amplitude Stage-I transport process filters candidate routes on a structural graph before a higher-cost Stage-II commitment step. In this manuscript, we model Stage-I only, using a mechanistically neutral GKSL open-system proxy with dephasing rate κ and diagonal disorder ε. The model does not imply microscopic quantum coherence in neural tissue. In two biological connectome benchmarks, selectivity improves under partial coherence. In a compact *Caenorhabditis elegans* touch-circuit benchmark, the wave proxy yields a 1.39 × improvement in peak target absorption over a matched low-temperature classical baseline. In a *Drosophila* larva mushroom-body motif (*N* = 243 active nodes), selectivity shows a pronounced non-monotonic disorder-enhanced selectivity peak at intermediate ε, strongest in the native topology and strongly attenuated by degree-preserving rewiring. A permutation-based reanalysis confirms the pre-specified DES contrast (ε = 3 versus ε = 0, *p* = 0.010), and the effect weakens progressively with increasing dephasing, becoming non-significant in the high-κ regime. We interpret these findings as evidence for a topology-sensitive, dephasing-dependent Stage-I routing effect on biological connectomes. Broader energetic and evolutionary implications remain conditional because Stage-II commitment is not explicitly modeled here.

## 1 Introduction

Neural computation faces energetic costs and information-theoretic lower bounds (e.g., Landauer’s bound; Landauer, 1961). Action potentials and synaptic transmission dominate the energetic budget of brain tissue — communication costs exceed computation costs by an order of magnitude (Lennie, 2003; Howarth et al., 2012; Levy and Calvert, 2021; Padamsey et al., 2022) — so additional computation is not free; search that relies on repeated spiking updates can become metabolically prohibitive at scale, as demonstrated experimentally under metabolic stress (Padamsey et al., 2022). This motivates an energy–information bottleneck: as hypothesis spaces grow (more states, more routes, more contingencies), purely diffusive exploration can become prohibitively slow — especially on bottlenecked or hierarchically structured graphs — while accelerating search by increasing spiking activity risks exceeding the available metabolic budget. On hierarchically structured graphs, this bottleneck is sharpened: a small number of bottleneck edges can dominate routing, so purely diffusive exploration can scale poorly as hypothesis spaces expand. Operationally, we use this term to denote a joint constraint on time and energy: purely diffusive exploration scales poorly in decision time on bottlenecked graphs, while brute-force spiking acceleration incurs high marginal metabolic cost.

The Free-Energy Principle (FEP) casts perception and action as approximate inference in which agents minimize a variational free energy bound on surprise (Friston, 2010). Classical attractor networks (e.g., Hopfield networks) show how recurrent connectivity can stabilize memories and decisions (Hopfield, 1982). Still, attractor convergence can be slow, and each update in a spiking substrate carries an energetic cost. In another domain, quantum and classical wave transport in noisy, disordered media has revealed a complementary principle: environmental noise can increase transport efficiency by preventing localization and enabling effective exploration (Plenio and Huelga, 2008; Mohseni et al., 2008; Caruso et al., 2009; Rebentrost et al., 2009; Engel et al., 2007; Cao et al., 2020). However, the extent to which such partially coherent transport regimes can be exploited as a decision primitive on realistic connectomes—and how this depends jointly on coherence and native topology for selectivity, not just throughput—remains underexplored. This motivates a complementary design question for computational neuroscience: can transient, wave-like propagation provide fast, low-cost exploration at the network level, while leaving final commitment to classical spiking dynamics?

We term this architectural principle Coherent-Resonant Netting (CRN) and emphasize that it does not posit microscopic quantum degrees of freedom. Stage-I is modeled as an effective (phenomenological) GKSL open-system proxy that is functionally equivalent to transient wave-like exploration, with dephasing rate κ controlling the interpolation between coherence and diffusion, while Stage-II remains a conceptual classical commitment layer. In this work, we simulate and measure Stage-I directly, and treat Stage-II as an abstract, high-cost commitment layer whose expected burden may be reduced when Stage-I concentrates probability mass on the target set.

Coherent-Resonant Netting’s distinctive signature is Disorder-Enhanced Selectivity (DES): a topology- and coherence-dependent improvement of benchmark selectivity under increasing diagonal disorder ε. In deeper decision architectures (e.g., the *Drosophila* mushroom-body motif) DES can be non-monotonic with a peak at intermediate ε, while shallower circuits can improve monotonically over the tested ε-range. CRN predicts that this is not a generic scalar-noise benefit: it should be topology-sensitive (native vs. degree-preserving rewiring) and bounded in κ, weakening outside an intermediate coherence window. Accordingly, the manuscript emphasizes negative controls and robustness audits rather than tuning parameters to a single circuit. Throughout, we operationalize decision selectivity using sink absorption ratios [Selectivity_end = P_T/(P_D + δ)], alongside coverage_end, Utility, and InfoPerCost (Methods 3.2; Supplementary Methods 1.4).

Objectives and outline. Here we formalize CRN’s Stage-I transport model and metrics, test the DES signature in two biological connectome benchmarks (*Caenorhabditis elegans* and a *Drosophila* larva mushroom-body decision motif), and use topology-destroying controls to isolate the coherence × topology interaction. We then derive falsifiable perturbational predictions that distinguish a dephasing-dependent transport effect from generic scalar-noise explanations. Exploratory mouse-proxy and evolutionary analyses are retained only as secondary context and are not used as primary evidence for the central claim.

## 2 The CRN framework

### 2.1 Two-factor mechanism: coherence × topology

Coherent-Resonant Netting predicts that decision efficiency depends on two coupled factors. In this work, we quantify decision efficiency primarily through selectivity and efficiency-per-cost metrics (Selectivity_end and InfoPerCost; Methods 3.2). Factor-I (“Coherence”) quantifies the extent to which propagation involves superposition rather than purely stochastic hopping. Factor-II (“Topology”) is the structural constraint imposed by the connectome, which shapes interference, localization, and spectral structure. In this view, the native connectome acts as an effective interference mask: it determines where interference is constructive vs. destructive, and thus whether transport concentrates on a target subspace (useful hypotheses/routes) or leaks to distractors.

We model Stage-I transport on a graph G(V, E) using a GKSL master equation for a density matrix ρ(t):


$$\begin{array}{c} \frac{d\,\rho }{d\,t}=-i\text{[}H,\rho ]+\kappa \underset{k}{\sum }(\Pi_{k}\rho \Pi_{k}-1/2\left\{\Pi_{k},\rho \right\})\\ +\sum_{s\in S}\eta_{s}\,\left(J_{s}\,\rho \,J_{s}^{\text{†}}-1/2\,\left\{J_{s}^{\text{†}}\,J_{s},\rho \right\}\right) \end{array}$$

Here, the structural graph provides the substrate for the Stage-I proxy: the symmetrized weighted connectivity enters the coherent generator through H_0_ = −γL_sym; κ controls dephasing toward more diffusive transport; Π_k ≡ | k><k| are site projectors in the pure-dephasing term; and J_s are sink jump operators attached to target and distractor node sets S = T ∪ D with couplings η_s. This formalism provides a continuous interpolation between more coherent transport and classical diffusion without implying a microscopic quantum substrate (Gorini et al., 1976; Lindblad, 1976; Whitfield et al., 2010; Kendon, 2007).

The baseline Hamiltonian is constructed from a symmetrized weighted Laplacian:


H⁢0=−γ⋅L⁢s⁢y⁢m

where L_sym = D_sym−W_sym and W_sym = (W + W*^T^*)/2.

Diagonal disorder is introduced as node-energy heterogeneity:


H=H⁢0+d⁢i⁢a⁢g⁢(E),w⁢i⁢t⁢h⁢E⁢_⁢i∼U⁢n⁢i⁢f⁢o⁢r⁢m⁢[−ε,+ε]

We treat ε as a phenomenological control parameter capturing heterogeneity in local excitability/thresholds; in biological contexts, such heterogeneity can be stress-related, but the mapping is context-dependent and not one-to-one. In disordered media, heterogeneity can induce Anderson localization, suppressing transport along many paths (Anderson, 1958). In networked decision motifs, this suppression can be constructive if it damps broad, high-entropy distractor pathways more strongly than the target subspace/pathways, producing DES.

### 2.2 Two-regime architecture: Stage-I netting and Stage-II fixation

Coherent-Resonant Netting is an architectural hypothesis about how biological agents allocate energy across computation. It distinguishes two regimes (or components) of decision processing that operate on different cost scales and may be implemented by different biophysical mechanisms.

Stage-I (“netting”, pre-selection) is a low-amplitude, subthreshold graded-flow regime that propagates activity over a fixed structural graph before global spiking commitment. Its computational role is to redistribute probability mass toward a reduced set of candidate hypotheses without modifying the graph itself. In this work, Stage-I is simulated explicitly: GKSL dynamics yield cumulative target and distractor sink captures P_T and P_D at the target and distractor sinks, and their values at T_end define Selectivity_end and coverage_end. Stage-II (“fixation,” commitment) is a high-gain, metabolically expensive regime that turns the filtered Stage-I outcome into a stable belief, memory, or action. In this work, Stage-II is not explicitly simulated; it remains architectural context rather than a modeled result.

Division of labor is motivated by a permeability paradox. For feed-forward routing problems (delivering a signal to a specific downstream target), higher permeability can be useful: wave-like dynamics can overcome bottlenecks and speed routing through noisy media. For recurrent associative memory, however, the same permeability can be harmful: if activity leaks too easily across weak inter-engram edges, false recall and spurious attractors increase. CRN therefore predicts that wave-like netting is advantageous for search-and-route motifs, whereas strong confinement and inhibitory gating are beneficial for stable attractor storage.

**FIGURE 1:**
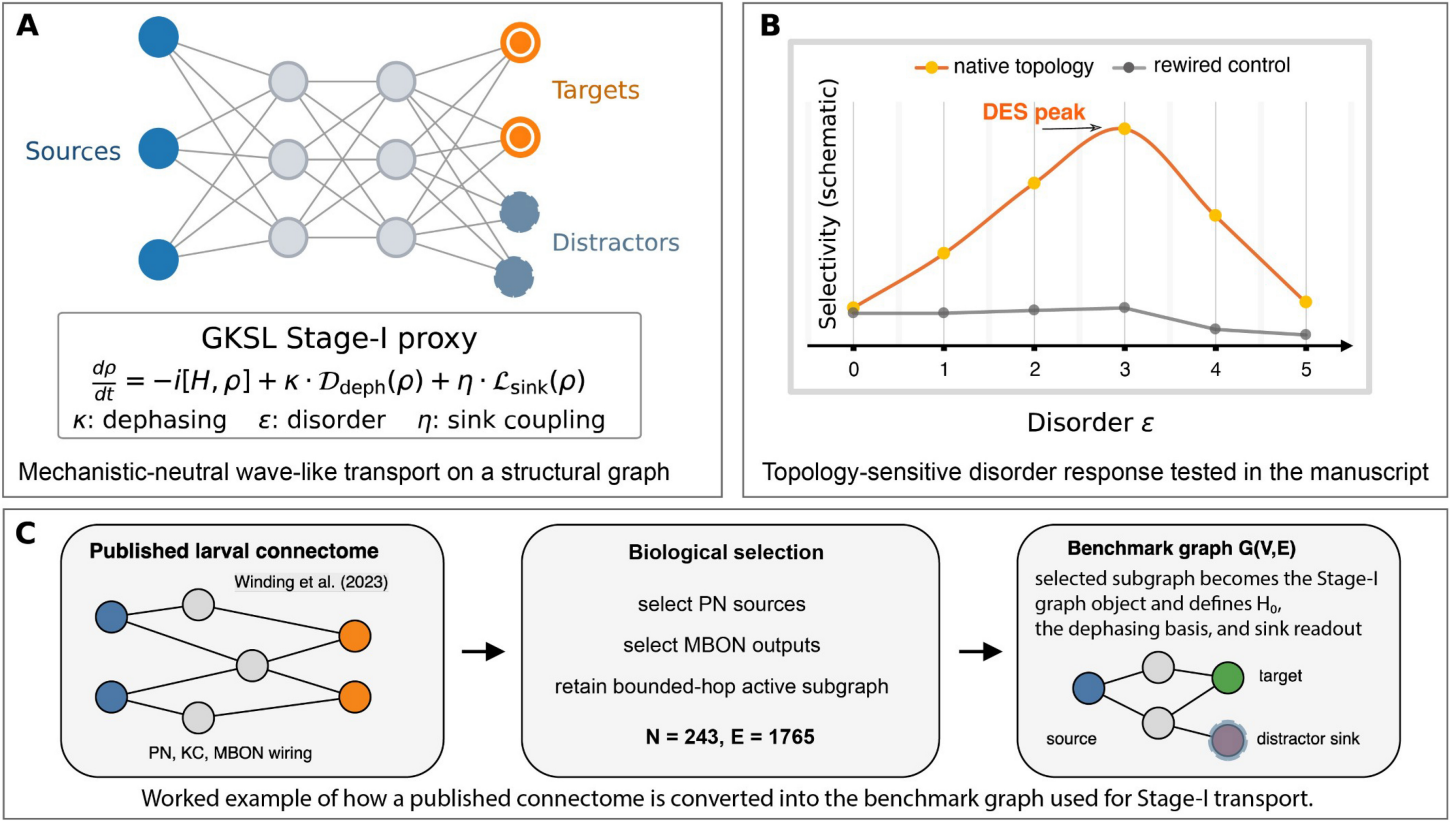
Coherent-Resonant Netting (CRN) architecture and the Stage-I transport signature. **(A)** Stage-I is represented by a mechanistically neutral GKSL transport proxy on a structural graph with dephasing κ, diagonal disorder ε, and absorbing target/distractor sinks. **(B)** The key signature tested in this manuscript is a topology-sensitive disorder response: native topology can support a Disorder-Enhanced Selectivity (DES) peak at intermediate ε, whereas degree-preserving rewiring attenuates the effect. **(C)** Worked *Drosophila* connectome-to-graph example: a published larval connectome excerpt is narrowed by biological selection (PN sources, MBON outputs, bounded-hop active subgraph) to produce the benchmark graph G(V,E); that selected subgraph then becomes the Stage-I graph object defining H_0_, the dephasing basis, and the target/distractor sink readout. Stage-II commitment/fixation is conceptual and is not simulated here.

**FIGURE 2:**
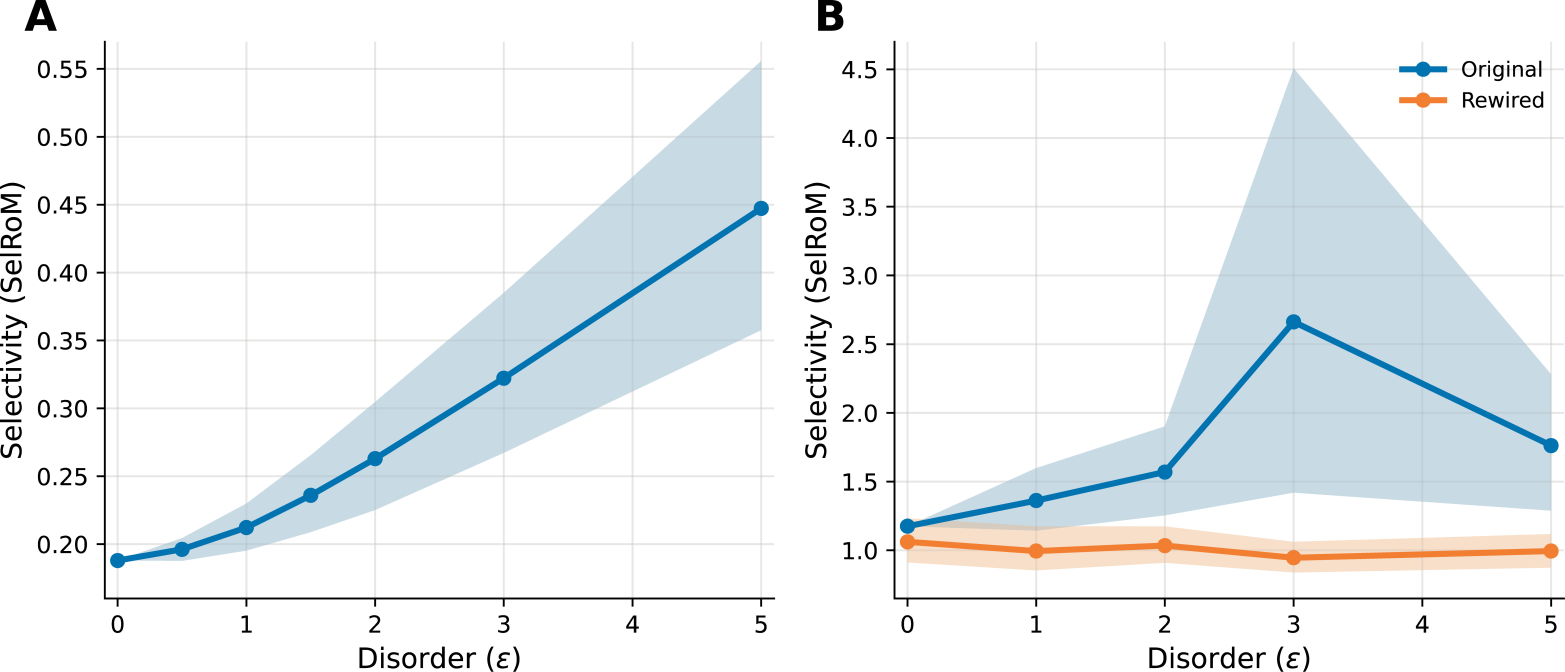
Disorder dependence of selectivity across biological connectome benchmarks. Selectivity ratio-of-means [SelRoM ≡ ⟨P_T⟩/⟨P_D**+δ** ⟩] versus diagonal disorder ε under the Stage-I transport proxy (κ = 0.001) for two biological benchmarks: **(A)** *C. elegans* touch circuit and **(B)** *Drosophila* larva mushroom-body motif (original vs. degree-preserving rewired control). Lines show means and shaded bands show 95% bootstrap confidence intervals across trial or replicate ensembles. The *Drosophila* panel shows a pronounced peak near ε ≈ 3 in the native topology.

Although we present this as Stage-I/Stage-II for intuition, the core claim is a two-factor interaction (coherence × topology): filtering and commitment need not be strictly sequential and may overlap in time. Conceptual schematics are provided in Figure 1 and Supplementary Figure 1; Figure 2 shows the two biological benchmarks, while Supplementary Figure 2 reports the broader cross-substrate summary including the exploratory mouse proxy.

A simple energy accounting clarifies the architectural motivation. Let EI be the energy cost of Stage-I netting and EII the marginal cost of one Stage-II commitment event (or a unit of high-gain spiking time). If Stage-I reduces the expected number or duration of commitments from Ncommit to Ncommit’, the net gain is ΔE ≈ (N_commit−N’_commit)⋅E_II−E_I. CRN predicts a net benefit when ΔE > 0, i.e., when the expected Stage-II savings exceed the Stage-I overhead. In the present paper this remains a conditional architectural implication rather than a demonstrated whole-cycle result, because Stage-I→Stage-II coupling is not explicitly simulated.

Energy budgets imply a strong asymmetry between spiking and subthreshold processing. We write C_spike = r⋅C_local, where C_spike is the cost of a spiking commitment cycle, and C_local is the cost of a unit of subthreshold processing; cortical estimates suggest r is O(10–100) (we use *r* ≈ 35 as a working value; Lennie, 2003; Howarth et al., 2012; Levy and Calvert, 2021; Padamsey et al., 2022; Attwell and Laughlin, 2001).

Under a repeat-until-correct toy assumption, the expected number of Stage-II commitment cycles scales as n_spike ∝ 1/P_T, where P_T denotes Stage-I target absorption. Then an α-fold increase in P_T reduces expected spike cycles by the same factor. While precise calibration is substrate-specific, the order-of-magnitude gap *r* ≫ 1 makes even modest Stage-I gains energetically meaningful when selection errors carry spike penalties (Supplementary Section 1.5).

As noted above, the permeability paradox motivates CRN’s division of labor: wave-like netting is suited to fast search-and-route motifs, whereas stable attractor storage benefits from confinement and inhibitory gating.

Finally, CRN is compatible with cognitome-level views in which cognition is organized not only by the static connectome but by a higher-order hypernetwork of cognitive elements and their integration dynamics (Anokhin, 2021). In CRN terms, Stage-I implements a fast proposal generator constrained by the effective interference mask of the connectome, while Stage-II selects and stabilizes a specific integrated configuration. The central empirical claim is then explicit: if native topology encodes useful interference structure, DES should be strongest in the native connectome and should weaken under topology-destroying controls.

### 2.3 Classical mimicry and mechanistic neutrality

A common concern is that GKSL implies a quantum substrate. We stress that this is not required for the architectural claim. Our claim concerns the functional regime: linear superposition-like propagation with tunable phase randomization operating on a fixed network topology, coupled to a classical commitment layer. A broad class of linear wave proxies with tunable damping and phase randomization can, in principle, exhibit interference and localization phenomena and thereby support non-monotonic regimes. GKSL is used here because it exposes two explicit control parameters (κ for dephasing and ε for disorder) and admits clear limiting baselines (wave-like vs. classical diffusion). Thus, failure of CRN predictions would falsify the architecture regardless of whether the underlying wave substrate is quantum or classical.

#### 2.3.1 Classical wave interpretation (mechanistic-neutrality note)

As a sanity check, in the limit κ→0 (negligible dephasing), our GKSL proxy reduces to coherent evolution on the graph, whereas for κ→∞ it approaches classical diffusion. This continuity ensures that our Stage-I model interpolates between wave-like and random-walk regimes without committing to a microscopic quantum substrate. In practice, mechanistic neutrality can be further probed by testing whether the same qualitative signatures (DES and its topology dependence under rewiring controls) persist under alternative linear wave proxies with matched damping.

## 3 Methods

We use GKSL/Lindblad dynamics as a mechanistic-neutral open-system wave proxy with tunable dephasing (κ) and diagonal disorder (ε), treated as phenomenological control parameters; this does not imply or require microscopic quantum coherence in neural tissue.

### 3.1 Model–substrate correspondence

The GKSL master equation acts on a density matrix ρ(t) defined over the nodes of a benchmark graph G(V,E). In this study, the graph is a structural benchmark graph: nodes are benchmark-defined anatomical units, edges are structural couplings derived from published connectome datasets, and sinks define target and distractor readouts. Table 1 summarizes the operational correspondence used in this manuscript, the level of abstraction at which it holds, and the principal non-claims of the model.

**TABLE 1:** Model-substrate correspondence for the Stage-I transport proxy.

Model element	Mathematical object	Operational mapping in this manuscript	Level of abstraction	What is not claimed
Node i in V	Basis state | i⟩	Benchmark node: an individually identified neuron or benchmark-specific anatomical unit	Structural graph node	Not a membrane-potential state, firing state, calcium trace, or BOLD time series
Edge weight w_*ij*_	Symmetrized connectivity W_sym entering H_0_ = −γ L_sym	Structural anatomical coupling in the benchmark graph	Structural connectivity	Not functional connectivity, effective connectivity, synaptic conductance, or voltage transfer
ρ_*ii*_(t)	Diagonal density-matrix element	Probability mass at node i under Stage-I transport dynamics	Transport occupancy proxy	Not neural activation or firing rate
P_*T*_(t), P_*D*_(t)	Cumulative absorption into target and distractor sinks	Stage-I target and distractor capture over a fixed horizon T_end	Readout score of transport	Not normalized end-state probabilities; residual mass may remain on the graph at T_end
κ	Dephasing strength in the Lindblad dephasing term	Phenomenological control of phase randomization / coherence loss in the transport proxy	Effective transport-regime parameter	Not a directly measured biophysical dephasing constant
ε	Diagonal disorder amplitude with E_*i*_ ∼ Uniform[−ε, +ε]	Phenomenological heterogeneity in local excitability / threshold landscape	Effective disorder parameter	Not temperature, and not a one-to-one stress biomarker
J_*s*_, η_*s*_	Sink jump operators and sink couplings	Absorbing readout channels attached to benchmark-defined target and distractor sets	Coarse-grained readout abstraction	Not a specific receptor mechanism, synapse type, or collapse postulate
Connectome	Benchmark graph G(V,E)	Structural wiring graph derived from published biological datasets	Anatomical graph abstraction	Not functional connectivity and not a protein-interaction network

A worked biological example is shown in Figure 1C. For the *Drosophila* larva benchmark, the published larval connectome of Winding et al. (2023) is reduced to the MB-centered active subgraph used in the main experiments (*N* = 243, E = 1,765). Figure 1C now makes that derivation explicit: a published larval connectome excerpt is narrowed by biological selection (PN sources, MBON outputs, bounded-hop active subgraph), and the resulting selected subgraph becomes the benchmark graph G(V,E) used for Stage-I transport. PN nodes define the source set, MBON nodes define 5 target and 5 distractor sinks, and that same selected subgraph defines H_0_, the dephasing basis, and the sink readout operators. Supplementary Section 2.2 gives the step-by-step derivation.

The graph enters the Stage-I equation through the symmetrized weighted Laplacian, so that the adjacency structure of the benchmark connectome defines the Hamiltonian generator H_0_ = −γL_sym. For the directed *Drosophila* benchmark, outgoing weights are first row-normalized to directed transition probabilities p(u→v) = w_uv/Σ_v w_uv and then symmetrized into the coupling matrix used to construct L_sym. The same vertex set defines the node-basis projectors used in the dephasing term, and designated target/distractor subsets of that graph define the sink jump operators J_s with couplings η_s. Thus, the Hamiltonian, dephasing basis, and readout operators are all defined directly on G(V,E).

The model therefore describes transport of probability mass on a fixed structural graph. It is not a direct simulation of neuronal spiking, membrane voltage, synaptic current, or macroscopic imaging signals. The GKSL formalism is used here as a mechanistically neutral Stage-I proxy because it provides a controlled interpolation between more coherent and more diffusive transport regimes on the same topology via κ, while ε perturbs that same topology through benchmark-defined diagonal heterogeneity. Stage-II remains conceptual in the present paper and is not explicitly simulated.

In the *Drosophila* benchmark, the graph is an active larval mushroom-body subgraph extracted from the connectome of Winding et al. (2023) with PN sources and MBON sink partitions (five targets and five distractors) retained within a bounded-hop benchmark graph. In the *C. elegans* benchmark, nodes correspond to individually named neurons in a compact touch-command-motor circuit derived from the canonical connectome, and target/distractor sinks are defined over motor outputs. The mouse case is explicitly a stochastic block-model proxy included for exploratory scale and task-class sensitivity analysis, not as an empirical mammalian micro-connectome.

### 3.2 Decision benchmark and metrics

Throughout, P_T and P_D denote cumulative absorption into target and distractor sink sets at the finite horizon T_end. They are readout measures of probability-flow capture, not normalized end-state probabilities; residual probability mass may remain on the graph at T_end. Accordingly, the model tracks transport of probability mass on a structural graph rather than direct neuronal activation. For each substrate, we define a target set T (desired routes/hypotheses) and a distractor set D (competing routes). Absorbing sinks attached to T and D yield cumulative finite-horizon absorption measures at T_end: P_T = P_sink,T(T_end) and P_D = P_sink,D(T_end). We define:


S⁢e⁢l⁢e⁢c⁢t⁢i⁢v⁢i⁢t⁢y⁢_⁢e⁢n⁢d=P⁢_⁢T/(P⁢_⁢D+δ),c⁢o⁢v⁢e⁢r⁢a⁢g⁢e⁢_⁢e⁢n⁢d=P⁢_⁢T+P⁢_⁢D.

Here δ = 10^∧^{−12} is used only to avoid undefined ratios. coverage_end = P_T + P_D serves as a throughput-proportional cost proxy for the Stage-I window.

To connect performance to energetic trade-offs, we define:


U⁢t⁢i⁢l⁢i⁢t⁢y⁢(λ)=P⁢_⁢T−λ⋅P⁢_⁢D

I⁢n⁢f⁢o⁢P⁢e⁢r⁢C⁢o⁢s⁢t=U⁢t⁢i⁢l⁢i⁢t⁢y⁢(λ)/(c⁢o⁢v⁢e⁢r⁢a⁢g⁢e⁢_⁢e⁢n⁢d+χ)

Unless stated otherwise, we use λ = 1 and χ = 0.01.

For architecture-dependence analyses, we also report:


P⁢_⁢g⁢o⁢o⁢d=P⁢r⁢(S⁢e⁢l⁢e⁢c⁢t⁢i⁢v⁢i⁢t⁢y⁢_⁢e⁢n⁢d>S⁢_⁢t⁢h⁢r⁢a⁢n⁢d⁢P⁢_⁢T>p⁢T⁢_⁢m⁢i⁢n).

We use S_thr = 2.0 and pT_min = 0.005 (Supplementary Methods 1.4).

Parameter sweeps over κ and ε identify regimes maximizing Selectivity_end or InfoPerCost under fixed T_end; when reporting “best-by-objective κ,” we display the upper envelope over κ at each ε (full 2D κ × ε maps are provided in the Supplementary materials).

### 3.3 Substrates and rationale

*C. elegan*s provides a uniquely complete connectome at single-synapse resolution and is therefore a minimal, high-credibility testbed for topology-dependent transport (Varshney et al., 2011). Its small size enables exhaustive parameter sweeps and mechanistic interpretation.

*Drosophila* larva mushroom body (MB) provides a canonical hierarchical decision motif: sparse expansion (PN→KC) followed by convergent readout (KC→MBON). It is an archetype for hypothesis selection in olfactory learning. We use the full larval connectome reported by Winding et al. and extract an MB active subgraph (extended selection; *N* = 243 nodes, 1,765 edges) by selecting PN sources (k_source = 10) and MBON sinks (k_target = 5 targets + 5 distractors) and retaining nodes/edges within a bounded hop distance (max_hops = 4) for benchmarking (Winding et al., 2023). Graph statistics and benchmark settings are summarized in Supplementary Tables 1, 2.

Mouse mesoscale connectome resources provide biological background for this proxy class (Oh et al., 2014), but the mouse case here is a stochastic block-model proxy and is not treated as an empirical mammalian micro-connectome.

### 3.4 Controls (competitors) and surrogate networks

To isolate topology dependence, we generate degree-preserving, type-aware rewired surrogates (Maslov–Sneppen randomization; preserving node classes/compartment labels; Maslov and Sneppen, 2002) and compare them to targeted pathway lesioning in the *Drosophila* MB motif (randomly dropping 50% of KC→MBON edges; lesion_drop = 0.5). For architecture-dependence experiments, we use n_trials = 20 independent disorder/energy draws per (ε, κ) condition on the native graph; for rewired_type and lesion_KC_MBON we use n_surrogates = 20 surrogate graphs per condition, each evaluated over n_trials = 20 draws (400 runs per (ε, κ) cell). As dynamical competitors, we include classical random walk (CRW) and thermally activated hopping (CRW_thermal) with Metropolis rates. Effects are evaluated over trial ensembles and (where applicable) over surrogate ensembles.

### 3.5 Simulation parameters

Table 2 summarizes the default numerical parameters used across substrates, unless otherwise stated (see Supplementary Methods 3 for details).

**TABLE 2:** Simulation parameters (default).

Parameter	Symbol	Default value	Notes
Coupling strength	γ	1.0	Normalized to adjacency / Laplacian scale
Sink absorption	η_sink	1.0	Strong absorption limit (fixed; distinct from node-energy disorder ε)
Time step	dt	0.05	Numerical stability verified (see Supplementary materials)
Integration time	T_end	10.0	At least 5 × relaxation time in tested graphs; mouse proxy uses T_end = 100 (Supplementary Table 10a).
Disorder range	ε	0–5	Exploratory sweep range used across substrates
Dephasing range	κ	0.001–10	Coherent to classical (high-κ / strong-dephasing) regime
Good-run threshold	S_thr, pT_min	2.0, 0.005	Used in P_good metric for robustness

Unless stated otherwise, all rates and disorder amplitudes are reported in normalized (dimensionless) units. In particular, κ (dephasing rate), ε (diagonal disorder amplitude), and T_env (temperature parameter of the classical baseline) are dimensionless under the model’s normalization (see section “3 Methods”).

### 3.6 Statistical analysis

Across all substrates, reported curves and optima are computed over ensembles of independent simulation runs (independent disorder draws and, where applicable, surrogate graphs). Uncertainty is summarized with percentile bootstrap confidence intervals computed over runs (*B* = 5,000 resamples unless otherwise stated; see Supplementary Methods 5 for substrate-specific ensemble sizes). For the *Drosophila* architecture-dependence benchmark at κ = 0.001, we use two-way ANOVA on run-level Selectivity_end and coverage_end to assess main effects of architecture variant and disorder level and to test departures from additivity across the full factorial design. ANOVA is therefore used here as a means-based omnibus model, not as the sole inferential basis for the specific DES claim. Because Selectivity_end is right-skewed and heteroscedastic, we also report residual diagnostics, effect sizes, log-transform robustness, and Kruskal-Wallis checks in the Supplement. To evaluate the specific DES claim more directly, we additionally use a distribution-free permutation test for the pre-specified contrast ε = 3 versus ε = 0 in the native *Drosophila* topology, and extend the same contrast across the κ sweep as a robustness analysis. As a distribution-respecting sensitivity check, we additionally fitted Gamma-family generalized linear models with a log link to the trial-level Selectivity_end values; this analysis is reported in Supplementary Table 8b.

The benchmark analyses in this study use explicit, inspectable numerical solvers on archived graph inputs; in the released *Drosophila* and *C. elegans* benchmark scripts, Stage-I transport is propagated with scipy.sparse.linalg.expm_multiply on the sink-augmented Liouvillian. The underlying benchmark datasets are archived as separate Zenodo records for the *C. elegans* touch circuit (10.5281/zenodo.18432680), the Drosophila larva mushroom-body benchmark is archived at 10.5281/zenodo.18697116 (Dolgikh, 2026a), and the mouse proxy artifacts (10.5281/zenodo.18433186), while the canonical CRN software snapshot is archived at 10.5281/zenodo.18338260. Random seeds are fixed and recorded for the stochastic analyses used in the revision-critical *Drosophila* permutation workflows. Reported figures and tables therefore trace to archived inputs and public scripts rather than to unverifiable chat output, and were reviewed by the author and a second human checker before inclusion in the manuscript and public archiving.

## 4 Results

### 4.1 Architecture overview

Figure 1 provides the conceptual anchor for CRN. Figure 1A defines the Stage-I GKSL proxy on a structural graph with tunable dephasing κ and disorder ε; Figure 1B shows the topology-sensitive disorder-response signature tested in this manuscript; and Figure 1C gives a worked connectome-to-graph example in the Drosophila larva benchmark, from a published larval connectome excerpt through biological selection to the benchmark graph G(V,E) used for Stage-I transport. The central mechanistic prediction tested below is a topology × dephasing interaction: native topology can support stronger target-selective routing than topology-destroying controls under partially coherent transport, with the strongest mechanistic test provided by the *Drosophila* MB motif.

### 4.2 Biological connectome evidence for disorder-sensitive Stage-I routing

Stage-I selectivity under the GKSL transport proxy improves with disorder in both biological benchmarks examined here, but the profile is circuit-dependent. Figure 2A provides the *C. elegans* monotone disorder-gain case, whereas Figure 2B isolates the Drosophila MB benchmark in which the native topology shows a pronounced non-monotonic peak near ε ≈ 3 and the degree-preserving rewired control is strongly attenuated. Figure 2 reports selectivity as the conservative ratio-of-means metric SelRoM versus ε at κ = 0.001.

In the *C. elegans* touch-circuit benchmark, SelRoM increases from 0.188 at ε = 0 to 0.447 at ε = 5 across the sweep shown. Stage-I also offers a throughput advantage in this benchmark: in a matched low-temperature classical baseline (T_env = 0.1), peak target absorption P_max improves from 0.716 (thermal) to 0.997 (wave proxy), a 1.39 × gain. Absolute SelRoM can be below 1 in this sink assignment because the distractor sink can absorb more strongly than the target; the key comparison is the disorder-induced gain relative to ε = 0.

### 4.3 *Drosophila* larva MB: a topology- and coherence-dependent DES peak

In the Drosophila larva mushroom-body connectome (*N* = 243 active nodes), Selectivity_end exhibits a robust non-monotonic dependence on disorder ε at low dephasing (κ = 0.001). In the native topology, run-level mean-of-ratios Selectivity_end increases from 1.18 at ε = 0 to 2.66 at ε = 3, then declines at ε = 5. The more conservative ratio-of-means SelRoM rises from 1.18 to 1.76 over the same contrast. As an omnibus model for the full architecture benchmark, two-way ANOVA shows main effects of VARIANT and EPSILON and a significant VARIANT × EPSILON departure from additivity (Table 3), but the DES claim itself is now anchored to the pre-specified permutation contrast reported below.

**TABLE 3:** Two-way ANOVA (κ = 0.001) on *Drosophila* MB architecture sweep.

Metric	Effect	*F*	df_1_	df_2_	*P*-value	η ^2^_p
Selectivity_end	VARIANT	44.70	2	4097	6.25e-20	0.021
Selectivity_end	EPSILON	31.12	4	4095	1.45e-25	0.030
Selectivity_end	VARIANT × EPSILON*	20.53	14	4085	4.16e-51	0.066
Coverage_end	VARIANT	14.33	2	4097	6.28e-07	0.007
Coverage_end	EPSILON	2906.98	4	4095	<1e-300	0.756
Coverage_end	VARIANT × EPSILON*	932.38	14	4085	<1e-300	0.762

*VARIANT × EPSILON denotes the full cell-means model (including interaction terms).

The DES peak is architecture-dependent. Degree-preserving rewiring markedly reduces the disorder benefit (bootstrap Δ(ε = 3-0) = +0.162 [0.056, 0.274]), whereas the native topology shows a much larger DES (Δ = +1.489 [0.249, 3.355]). Target-pathway lesioning attenuates but does not eliminate DES (Δ = +1.102 [0.830, 1.398]). Between-variant bootstraps confirm that DES in the native topology exceeds rewired surrogates (ΔDES = +1.328 [0.069, 3.232], *p* < 0.001), supporting the claim that the effect is not explained by degree distribution alone.

Because the DES claim concerns a specific intermediate-disorder contrast rather than a monotonic shift across all ε levels, we complemented the omnibus analysis with a distribution-free permutation test for the pre-specified contrast ε = 3 versus ε = 0. In the primary *Drosophila* architecture benchmark at κ = 0.001, this contrast is significant (Δ = 1.487, p_perm = 0.010, *n* = 20). We then extended the same contrast across a companion κ-sweep benchmark on a separate robustness branch. The effect weakens progressively with increasing dephasing (Δ = 2.349 at κ = 0.001, 0.685 at κ = 1.0, 0.284 at κ = 3.0, and 0.053 at κ = 10.0), with significance lost in the high-κ regime (p_perm = 0.203 at κ = 10.0). We interpret this as dephasing-dependent attenuation of DES, consistent with a transport-regime-specific effect rather than a generic scalar-noise benefit.

### 4.4 Architecture dependence across κ

**FIGURE 3:**
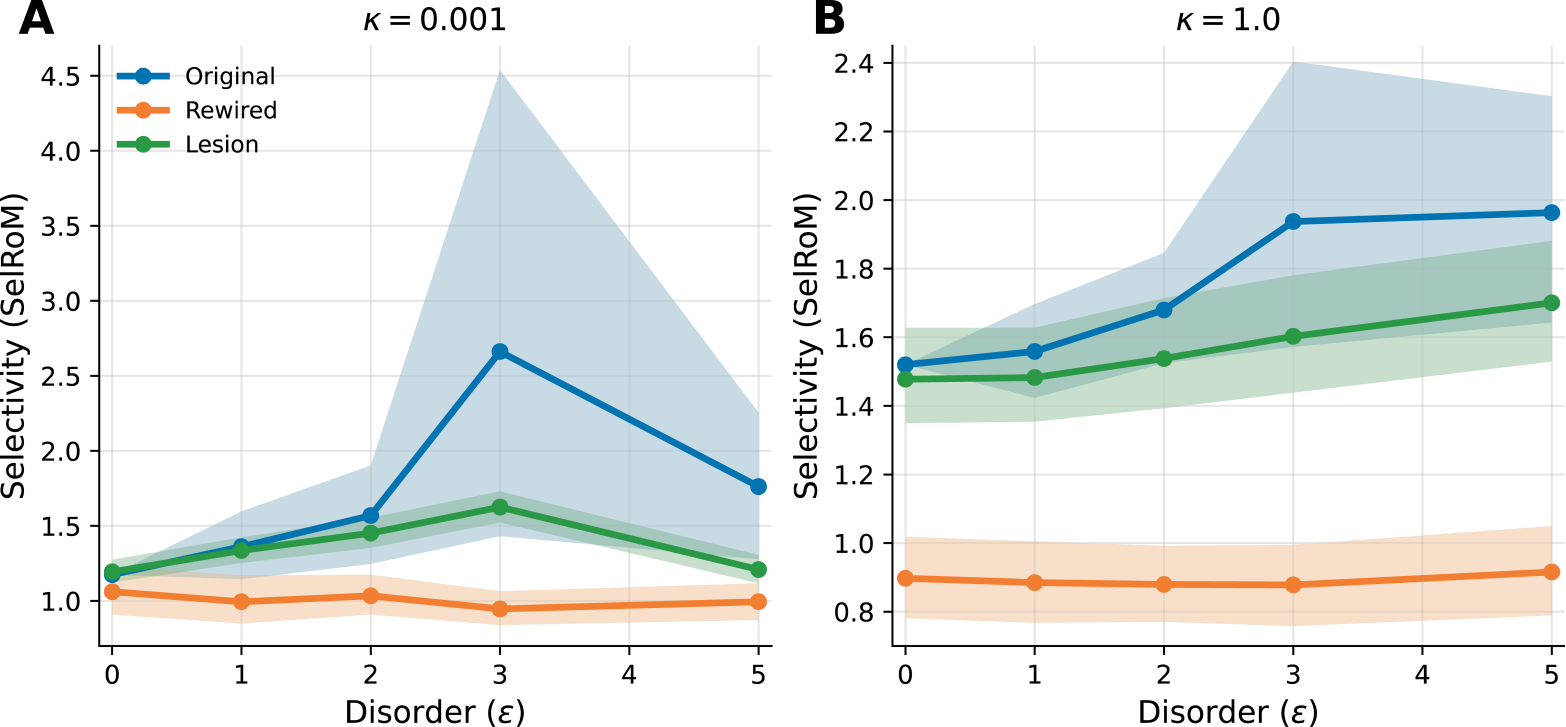
Architecture dependence of Disorder-Enhanced Selectivity (DES) in the *Drosophila* larva mushroom body. **(A)** κ = 0.001 (low dephasing) and **(B)** κ = 1.0 (higher dephasing). Selectivity is shown as a ratio-of-means across replicate means for original, rewired control, and lesion variant. Shaded bands show 95% bootstrap confidence intervals across replicate ensembles. At κ = 0.001, the original topology exhibits a pronounced non-monotonic peak near intermediate ε; this peak is strongly attenuated under rewiring and partially reduced by lesioning. The revised statistics additionally support the native ε = 3 versus ε = 0 contrast by permutation, and ε-sensitivity is reduced under stronger dephasing.

Figure 3 resolves the Drosophila architecture comparison across dephasing levels: Figure 3A shows the low-κ regime where the native topology exhibits the clearest intermediate-ε peak, and Figure 3B shows the higher-κ regime where the curves flatten and ε-sensitivity is reduced. The same ε = 3 versus ε = 0 contrast weakens progressively across the κ sweep, becoming only borderline at κ = 3.0 and non-significant in the high-κ regime at κ = 10.0 (Supplementary Table 8a and Supplementary Figure 15a). At κ = 1.0, the profiles become more monotone and ε-sensitivity is reduced relative to κ = 0.001, although a topology-dependent ordering persists.

This comparison also makes the manuscript’s boundary claims explicit. Optimization axis: the effect is defined on diagonal disorder ε on a fixed structural graph, not on scalar noise intensity in a threshold device. Coherence dependence: the ε = 3 versus ε = 0 contrast weakens as κ increases and becomes non-significant in the high-κ regime. Topology dependence: degree-preserving rewiring strongly attenuates the low-κ peak.

To complement mean Selectivity_end, we report P_good, the fraction of runs that meet the selectivity-and-absorption criterion (Selectivity_end > S_thr and P_T > pT_min; Methods 3.2; Supplementary Methods 1.4). P_good shows an intermediate-ε enhancement in the native and lesion variants and is strongly suppressed by rewiring (Supplementary Figures 11, 14); coverage_end declines at high disorder as expected (Supplementary Figures 12, 15).

### 4.5 Energy-aware objectives and information-per-cost

Energy-aware objectives provide a secondary consistency check rather than primary evidence. In the Drosophila benchmark, both InfoPerCost and Utility can exhibit an intermediate-disorder optimum under partially coherent GKSL transport. At higher disorder, the low-temperature thermal baseline approaches GKSL values, consistent with disorder dominating over coherence effects. Full objective curves and κ × ε sweeps are reported in the Supplementary materials.

## 5 Falsifiability and comparison

### 5.1 Competitors and distinctive features

Coherent-Resonant Netting is intended as a mechanistically neutral Stage-I transport framework rather than a universal replacement for existing theories of neural inference and decision-making. The closest alternatives operate at different explanatory levels, including predictive coding (Rao and Ballard, 1999), active inference / the Free-Energy Principle (Friston, 2010), classical noise-benefit accounts (Gammaitoni et al., 1998; McDonnell and Ward, 2011), diffusion-decision models (Ratcliff and McKoon, 2008), connectome communication models (Goñi et al., 2014), and connectome-harmonic / spectral models (Atasoy et al., 2016). Table 4 summarizes the main overlaps, distinctive claims, and current limitations of CRN relative to these neighboring frameworks.

**TABLE 4:** Positioning Coherent-Resonant Netting (CRN) relative to neighboring frameworks.

Framework	Primary explanatory level	Core mechanism	Where it overlaps with CRN	What CRN adds or changes	What CRN does not yet provide
Predictive coding	Algorithmic / circuit-level inference	Iterative error minimization over hierarchical message passing	Both address selective hypothesis filtering before stable interpretation	CRN proposes a topology-constrained Stage-I transport substrate for rapid pre-commitment routing	No explicit error neurons or full hierarchical message-passing architecture
Free-energy principle / active inference	Normative inference principle	Approximate belief updating that minimizes variational free energy or prediction error	Both are motivated by efficient inference under resource constraints	CRN offers a transport-level proposal for candidate-hypothesis filtering on a structural graph	No generative model, posterior beliefs, policy inference, or variational objective
Classical noise-benefit frameworks	Dynamical systems / signal-detection level	Added noise improves detection or threshold crossing in non-linear systems	Both can generate intermediate-regime performance benefits	CRN predicts a dephasing-dependent and topology-sensitive transport effect with attenuation under rewiring	Not a threshold-crossing detector model and not reducible to a generic scalar-noise benefit
Diffusion-decision / race / attractor commitment models	Commitment and action-selection level	Evidence accumulation or attractor competition leading to a categorical choice	CRN shares the idea that decision-making includes a final commitment stage	CRN places these models downstream as Stage-II fixation / commitment mechanisms	The present manuscript does not yet simulate Stage-II jointly with Stage-I
Connectome communication models	Structure-constrained communication level	Navigation, communicability, diffusion, search information, or related graph communication scores	Both ask how structural topology shapes route selection or communication efficiency	CRN adds open-system dephasing, disorder, and explicit sink readouts on the same structural graph	No claim that communication models are replaced; they remain important classical baselines
Connectome harmonics / spectral models	Whole-network propagation / spectral geometry	Propagation is described through connectome eigenmodes or related spectral decompositions	Both treat connectome topology as a propagation substrate	CRN adds finite-horizon target/distractor readouts and tunable dephasing + disorder	No whole-brain harmonic decomposition or direct bridge to functional harmonic analysis

These frameworks are best read as complementary rather than adversarial. Predictive coding and Free-Energy / Active-Inference accounts remain higher-level descriptions of inference; diffusion-decision, race, and attractor models remain natural candidates for downstream commitment; connectome communication and harmonic models remain important baselines for structure-constrained propagation. The present contribution is narrower: on a fixed structural graph, partially coherent transport with tunable dephasing and diagonal disorder can generate topology-sensitive Stage-I routing signatures, most clearly the Drosophila DES effect, that are not captured by purely diffusive or purely scalar-noise descriptions alone.

### 5.2 Falsifiable predictions

P1 (stress curve): In decision motifs analogous to the mushroom body, moderate metabolic stress (such as standard food-deprivation protocol (e.g., 12–24 h) or temperature perturbation) should improve behavioral selectivity relative to both homeostatic and severe-stress conditions, producing an inverted-U curve (Yerkes and Dodson, 1908). Effects should be strongest in tasks that tax hypothesis selection rather than long-term storage.

P2 (topology dependence): Genetic or developmental manipulations that simplify MB topology (reduced KC connectivity, disrupted compartmentalization) should flatten the stress curve. In silico degree-preserving rewiring predicts strong attenuation; the biological analog is reduced motif specificity.

P3 (anesthesia / dephasing scan): If anesthesia may act as an effective dephasing agent for mesoscale wave dynamics, increasing anesthetic depth should reduce DES and suppress intermediate-ε benefits. Temperature scans (e.g., 18 °C–30 °C for *Drosophila*) should reveal non-monotonic “windows” where selectivity peaks, consistent with predictions of coherence-time sensitivity.

P4 (noise injection without stress): Weak, uncorrelated subthreshold perturbations (e.g., optogenetic “shimmering”) in a non-stressed preparation should reproduce intermediate-ε benefits without actual nutrient deprivation, if ε primarily reflects excitability heterogeneity.

P5 (motif specificity/permeability paradox): In recurrent attractor-storage motifs, increasing permeability via Stage-I-like dynamics should increase false positives. Therefore, the direction of the stress effect should depend on circuit class: search-and-route motifs show DES, storage motifs show permeability-paradox degradation.

Each prediction is falsifiable: absence of an inverted-U in appropriate decision motifs, lack of topology sensitivity, or monotone effects of anesthetic/temperature scans would jointly undermine the CRN architectural claim.

## 6 Discussion and conclusion

On biologically grounded structural graphs, a mechanistically neutral Stage-I transport proxy can generate topology-sensitive, dephasing-dependent selective-routing signatures. The strongest evidence is the native-vs.-rewired Drosophila MB DES effect, complemented by a *C. elegans* throughput advantage over a matched low-temperature classical baseline. Exploratory mouse-proxy and evolutionary analyses are retained in the Supplementary materials and are not used as primary evidence for the central claim.

Stage-I does not implement structural plasticity or Hebbian learning. The graph topology is fixed throughout each simulation: no edges are added, removed, or reweighted. Accordingly, the “pruning” discussed here is dynamical rather than structural. For a fixed graph and fixed target/distractor sink partition, the Stage-I transport dynamics can increase the target-distractor absorption contrast P_T/(P_D + δ) by routing probability mass more strongly toward target sinks than toward distractors. In this sense, Stage-I narrows the effective hypothesis set by differential routing on a fixed substrate, not by modifying the substrate itself.

The energy-information bottleneck motivates why a low-cost filtering stage would be useful, but the present paper does not claim to demonstrate a full whole-cycle energetic benefit. What is demonstrated here is narrower: under a mechanistically neutral Stage-I transport proxy, biological connectome motifs can exhibit target-biased routing and, in the *Drosophila* benchmark, a topology-dependent disorder-enhanced selectivity peak under partial coherence. Any whole-cycle energetic advantage depends on how Stage-I outputs are converted into Stage-II commitment dynamics. Because Stage-II is not explicitly modeled here, that consequence remains conditional rather than demonstrated.

Stage-II (commitment/fixation) is not simulated in this manuscript. References to fixation, commitment, or broadcast are architectural context rather than modeled results. The direct claims of the present paper concern only Stage-I transport outputs and their dependence on topology, disorder, and dephasing. We therefore interpret the present results as establishing a necessary Stage-I property for a two-stage solution, not as a full demonstration of whole-cycle energetic benefit.

In the *Drosophila* benchmark, the disorder contrast is strongest at low dephasing, weakens progressively as κ increases, and becomes non-significant in the high-κ regime tested here. Degree-preserving rewiring also strongly attenuates the effect. We therefore interpret DES as a dephasing-dependent, topology-sensitive transport phenomenon rather than as a generic scalar-noise benefit.

These three labels summarize the present claim boundary in plain terms: Optimization axis, Coherence dependence, and Topology dependence. The manuscript now uses those labels directly so the Drosophila DES result is not framed as a generic stochastic-resonance effect.

### 6.1 Limitations

Several limitations constrain the interpretation of our results:

Model simplifications: The GKSL proxy assumes linear dynamics with instantaneous sinks. Real neural circuits exhibit non-linear gain functions, dendritic compartmentalization, finite synaptic time constants, and adaptive inhibition. Whether DES persists under these extensions requires explicit modeling. The GKSL proxy is also Markovian (memoryless), so history-dependent (non-Markovian) effects are not captured in the present formulation.

Biophysical substrate ambiguity: We demonstrate that a wave-like substrate with tunable dephasing can produce DES, but we do not identify the biophysical mechanism that implements Factor-I (coherence) *in vivo*. Candidates include subthreshold dendritic oscillations, gap-junction networks, ephaptic coupling, and fast inhibitory sculpting, each with distinct spatial/temporal signatures.

Parameter-to-biology mapping: We treat disorder ε as a phenomenological proxy for heterogeneity in local excitability/energy levels; such heterogeneity can be stress-related, but it may also reflect other physiological knobs (temperature, neuromodulation, ionic gradients, synaptic release probability), and the mapping is not one-to-one. Experimental calibration is required to translate ε and κ into measurable perturbations.

Envelope reporting: Best-by-objective “envelopes” (max over κ at each ε) summarize achievable regimes but can obscure multi-modal κ dependence; for transparency, we provide full κ × ε maps and boundedness checks across κ in the Supplement.

Selectivity_end is a right-skewed ratio metric for which both normality and homoscedasticity are violated in the native *Drosophila* benchmark. We therefore treat ANOVA as a means-based omnibus model rather than as the sole inferential basis for DES. The revision adds a distribution-free permutation test for the pre-specified ε = 3 versus ε = 0 contrast and a κ-gradient robustness analysis; together these support a dephasing-dependent Drosophila DES effect even though the original native-topology-only Kruskal-Wallis omnibus test is borderline (Supplementary Section 5, Supplementary Table 8a, Supplementary Figure 15a).

Parameter sensitivity and topology dependence: The DES peak location and magnitude are topology- and sink-placement-dependent. Generalization to other circuits requires substrate-specific calibration and negative-control tests (rewiring, lesions, or targeted motif disruption).

No closed-loop validation. Stage-I outputs are not coupled to a realistic Stage-II spiking or attractor model in this work. The energy-savings argument remains assumption-bound until validated with explicit Stage-I/Stage-II simulations that track both information gain and metabolic cost through a complete decision cycle.

Scale gap in the mammalian case: The largest substrate in the present study remains a stochastic block-model mouse proxy rather than an empirical mammalian micro-connectome at synapse-level resolution. We therefore treat the mouse analysis as an exploratory boundary case rather than as core biological evidence, and any broader evolutionary interpretation remains supplementary and explicitly provisional.

Exploratory mouse-proxy and evolutionary analyses are reported in the Supplementary materials and are not used as primary evidence for the central Stage-I claim (Dolgikh, 2026b).

## Data Availability

Datasets and supplementary exploratory artifacts are archived on Zenodo: auxiliary evolutionary game-theory dataset (https://doi.org/10.5281/zenodo.18379850), *C. elegans* touch-circuit artifacts (https://doi.org/10.5281/zenodo.18432680), *Drosophila* larva mushroom-body benchmark artifacts (https://doi.org/10.5281/zenodo.18697116), and mouse proxy artifacts (https://doi.org/10.5281/zenodo.18433186). The code used in this study is archived on Zenodo: https://doi.org/10.5281/zenodo.18338260.
