# Supplementary material for: Coherent-resonant netting: disorder-enhanced selectivity from transient wave-like dynamics on biological connectomes
Source: Front Comput Neurosci. 2026 May 13;20:1813959. doi: 10.3389/fncom.2026.1813959 (PMC13212248; doi:10.3389/fncom.2026.1813959)
Supplement: Supplementary file 1 [file Data_Sheet_1.pdf]

SUPPLEMENTARY MATERIALS

Scope. This Supplement provides: (i) dataset provenance; (ii) formal metric definitions and notation; (iii) complete κ×ε sweep results; (iv) robustness checks and controls; and (v) extended figures and tables referenced in the Core. Conceptual motivation and architectural claims are presented in the Core (Sections 2.1–2.3).

Contents:

S1. Mathematical specification of CRN
S2. Datasets and graph construction
S3. Simulation protocol and metrics
S4. Baselines and negative controls
S5. Statistical analysis
Supplementary Results
S6. Disorder sensitivity in connectome benchmarks (summary figures)
S7. *Drosophila* larva connectome benchmark
S8. Localization diagnostics
S9. Architecture dependence and negative controls
S10. Mouse cortical proxy: energy–selectivity trade-off
S11. Evolutionary game-theoretic selection for modular regime separation
Supplementary References

Supplementary Methods

S1. Mathematical specification of CRN

CRN (Coherent Resonant Netting) is defined as a two-regime decision architecture (conceptually Stage-I/Stage-II). Stage-I performs a low-amplitude, wave-like exploration that prunes a hypothesis space at low marginal cost, and Stage-II corresponds to a high-gain commitment/readout mechanism (e.g., spiking fixation) that acts on the pruned set. In the Supplementary Materials, we formalize Stage-I as an open-system wave proxy and evaluate its functional outputs by measuring absorption into Target vs. Distractor sinks.

S1.1 Graph and state space

In simulations, we define the absorption score (target absorption) as the expected occupancy of the sink node i at T_end, scaled by the number of vertices |V| and sink coupling w_ij:

$$A = |V| \cdot w\_ij \cdot \rho\_ii(t\_end)$$

Baseline Hamiltonian H_0 from a symmetrized weighted Laplacian, where L_sym = D_sym - W_sym and W_sym = (W + W^T)/2:

$$H0 = -\gamma \cdot Lsym.$$

S1.2 Open-system dynamics (GKSL proxy)

Stage-I is modeled with the GKSL master equation with dephasing and absorbing sinks. GKSL is used as a functional proxy for transient wave dynamics with tunable decoherence/measurement, not as a claim of microscopic quantum coherence in neural tissue. GKSL evolution is Markovian (memoryless), providing a controlled interpolation between coherent and diffusive limits; non-Markovian extensions are left for future work.

$$\frac{d\rho}{dt} \&= -i[H,\rho] + \kappa \sum_k \left(\Pi_k \rho \Pi_k - 1/2\,\{\Pi_k, \rho\}\right)$$

$$\&+ \sum_{s \in S} \eta_s \left(J_s \rho J_s^\dagger - 1/2\,\{J_s^\dagger J_s, \rho\}\right)$$

Here, Π_k ≡ |k⟩⟨k| is the site-basis projector (pure dephasing term).

κ controls the coherence–measurement balance (κ→0: coherent exploration; κ≫1: strongly dephased, more classical transport). Sinks are implemented using jump operators Js that remove population from designated nodes and direct it to external accumulators (Target vs Distractor).

S1.3 Disorder parameter ε

Energetic heterogeneity is modeled as diagonal disorder:

$$H = H0 + diag(E), with\ E\_i \sim Uniform[-\varepsilon, +\varepsilon].$$

We treat ε as a phenomenological control parameter for heterogeneity in local excitability/energy levels; any interpretation in terms of stress or physiology is context-dependent.

S1.4 Metrics

At T_end we report sink absorption probabilities P_T (Target) and P_D (Distractor) (P_T = P_sink,T(T_end), P_D = P_sink,D(T_end)), and define:

$$Selectivity\_end = P\_T\ /\ (P\_D + \delta), coverage\_end = P\_T + P\_D.$$

We report both a utility function and an information-per-cost metric:

$$Utility(\lambda) = P\_T - \lambda \cdot P\_D$$

$$InfoPerCost = Utility(\lambda)\ /\ (coverage\_end + \chi)$$

$$P\_good = Pr(Selectivity\_end > S\_thr\ \wedge\ P\_T > pT\_min).$$

Unless stated otherwise: λ=1, χ=0.01, S_thr=2.0, pT_min=0.005.

S1.5 Energetic scaling (why modest Stage-I gains matter)

To connect selectivity gains to energetic impact, we use the empirical asymmetry between suprathreshold spiking and subthreshold integration (Lennie, 2003; Howarth et al., 2012; Levy and Calvert, 2021; Padamsey et al., 2022), confirmed by updated energy audits showing that postsynaptic signaling dominates cortical energy use (Howarth et al., 2012) and that communication consumes ~35× more energy than computation (Levy and Calvert, 2021). We use r ≈ 35 as a working value; recent estimates suggest this may be conservative (Levy and Calvert, 2021). Let C_spike be the metabolic cost of a spiking commitment cycle and C_local the cost of a unit of subthreshold processing; write C_spike = r·C_local with r≫1 (r≈35 used for illustration). In a repeat-until-correct toy loop, the expected number of costly Stage-II cycles scales as n_spike ∝ 1/P_T, where P_T is the probability of a correct commitment after Stage-I filtering. If filtering increases P_T by a factor α, then expected spike-cycle count decreases by 1/α; thus, α=1.39 implies ≈28% fewer costly spike cycles. With r≈35, this corresponds to ≈0.28×35≈10 local-processing units per avoided cycle, so even modest Stage-I improvements can yield disproportionate metabolic savings when selection errors carry spike penalties. This back-of-the-envelope scaling is illustrative: the simulations do not explicitly model Stage-II spike counts, so r and the mapping from P_T to the number of behavioral commitment cycles should be treated as substrate- and protocol-dependent parameters rather than calibrated predictions.

S2. Datasets and graph construction

S2.1 *C. elegans* touch circuit

A compact C. elegans touch-withdrawal benchmark (N=24) is assembled as an abstracted touch→command→motor circuit derived from the Varshney et al. connectome (Varshney et al., 2011). The node set is partitioned into 6 touch sensory sources {ALML, ALMR, AVM, PLML, PLMR, PVM}, 4 command interneurons {AVD, PVC, AVA, AVB}, and 14 motor outputs {VA1–VA4, VB1–VB4, DA1–DA3, DB1–DB3}. The graph is an undirected weighted union of canonical touch-circuit chemical synapses (weight 1) and gap junctions (weight 2), plus a weak chain coupling between adjacent motor nodes (weight 0.2) to enforce a realistic motor manifold. For the selectivity experiments (Core Figure 2A; κ=0.001), three motor nodes {VA1, VB1, DA1} are designated as targets and the remaining motor nodes serve as distractor sinks.

S2.2 *Drosophila* larva mushroom body (Winding et al., 2023)

A directed weighted graph is built from the larval Drosophila connectome (Winding et al., 2023). Before constructing the Stage-I Hamiltonian, outgoing weights are row-normalized to directed

transition probabilities p(u→v)=w_uv/∑_v w_uv and then symmetrized into the weighted matrix used to build W_sym and L_sym. We extract an MB-centered subgraph and an active subgraph connecting selected PN sources to MBON sinks.
Core Figure 1C shows the same derivation as a worked schematic. Starting from a published larval connectome excerpt, we perform the biological selection step used in the benchmark: select PN sources, select MBON outputs, and retain the MB-centered active subgraph within the benchmark hop bound. The resulting selected subgraph is the benchmark graph G(V,E) used in the main experiments. In the revision-critical benchmark this gives N=243 nodes and E=1765 edges.
Operationally, the selected subgraph then becomes the Stage-I graph object: outgoing weights are row-normalized to directed transition probabilities p(u→v), the directed matrix is symmetrized to W_sym to define L_sym and H_0, the retained PN nodes initialize the source distribution, and the retained MBON nodes are partitioned into 5 target and 5 distractor sinks. Thus the connectome, the graph G(V,E), and the readout structure remain explicitly linked.

Supplementary Table S1. Drosophila graph sizes used in the MB benchmark. The active subgraph (extended; N=243, E=1765) is used in all Drosophila benchmark figures and tables unless stated otherwise.

Graph	nodes	edges
**core MB subgraph**	379	2137
**extended MB subgraph**	553	3875
**active subgraph (core)**	80	381
**active subgraph (extended)**	243	1765

Extended-graph benchmark selection sizes: PN sources=10, MBON targets=5, MBON distractors=5.

S2.3 Mouse cortical proxy (SBM)

A stochastic block model (SBM) of three recurrent assemblies is used as an exploratory mouse cortical proxy to probe scalability and the permeability-selectivity trade-off. The SBM uses N=150 nodes partitioned into 3 blocks (50 nodes each), with within-block connection probability pin=0.30 and between-block probability pout=0.02; edges are undirected (symmetric) in the transport proxy. In addition to the temperature/permeability scans over T_env∈{0.1, 0.3, 1.0, 3.0} at fixed ε=3 (Tables S9-S10; Figure S16), we run a disorder sweep ε∈{0,1,2,3,4,5,8} at fixed T_env=0.1, summarized in Table S10a and reproduced in Supplementary Figure S2C.

S3. Simulation protocol and default parameters

Unless stated otherwise, we use γ=1.0, η_sink=1.0, dt=0.05, T_end=10.0. Each condition is evaluated over multiple disorder draws and, where applicable, multiple surrogate graphs.

Supplementary Table S2. Key numerical parameters (Drosophila benchmark config).

parameter	value
**gamma**	1.0
η_sink	1.0
**dt**	0.05
**T_end**	10.0
**κ_grid**	0.001, 0.003, 0.01, 0.03, 0.1, 0.3, 1.0, 3.0, 10.0

S4. Baselines and negative controls

Baselines include a classical random walk (CRW) and thermal random walks in the same energy landscape. The main biological-benchmark comparisons report $T\_env \in \{0.1, 1.0\}$; the exploratory mouse proxy additionally scans $T\_env \in \{0.3, 3.0\}$.

S5. Statistical analysis

Across the revision-critical Drosophila architecture benchmark, ANOVA is retained as a means-based omnibus model for the full factorial design, but the specific DES claim is evaluated with a distribution-free permutation contrast for $\varepsilon=3$ versus $\varepsilon=0$ in the native topology.

Bootstrap confidence intervals are computed with the percentile method over simulation runs (B=5000 resamples; fixed random seed), unless stated otherwise. The primary native-topology architecture contrast at $\kappa=0.001$ is reported in the text above. Supplementary Table S8a now begins with that same primary A8 architecture benchmark row ($\Delta=1.487$, p_perm=0.010, n=20) and then lists the companion A7 κ-sweep rows (n=10 per κ) that re-evaluate the same $\varepsilon=3$ versus $\varepsilon=0$ contrast across κ on a distinct native-topology run family.

The original native-topology-only omnibus tests are borderline (ANOVA $p=0.095$; Kruskal–Wallis $p=0.098$) because the DES signal is concentrated at the intermediate $\varepsilon=3$ level rather than as a monotonic shift across all five ε values. In the primary architecture benchmark at $\kappa=0.001$, the permutation contrast is significant ($\Delta=1.487$, p_perm=0.010, n=20). The companion κ-sweep rows in Table S8a are separate robustness results with n=10 per κ level; their $\kappa=0.001$ entry ($\Delta=2.349$, p_perm=0.008) is therefore not a replacement for the primary architecture estimate. Across that companion sweep, the contrast attenuates with κ and becomes non-significant in the high-κ regime at $\kappa=10.0$.

S5.1. ANOVA diagnostics and effect sizes

Partial eta-squared ($\eta^2 p$) and omega-squared (ω^2) are reported for each ANOVA term (Supplementary Table S5a). For Selectivity_end, $\eta^2 p$ values are small, reflecting high within-cell variance from disorder sampling; the DES signal manifests primarily as an upper-tail shift at intermediate ε rather than as a monotonic location shift across all ε levels.

Both normality and homoscedasticity are violated for Selectivity_end. We therefore retain ANOVA and Kruskal-Wallis as conservative omnibus context and use permutation plus bootstrap as the primary support for the DES claim in the revised manuscript.

Supplementary Table S5a. ANOVA extended

Metric	Effect	SS	df_effect	df_residual	F	eta_sq_partial	omega_sq
Selectivity_end	VARIANT	186.453875	2	4085	46.68	0.0223	0.0209
Selectivity_end	EPSILON	257.586675	4	4085	32.25	0.0306	0.0286
Selectivity_end	VARIANT × EPSILON	129.923415	8	4085	8.13	0.0157	0.0130
coverage_end	VARIANT	0.002268	2	4085	59.51	0.0283	0.0068
coverage_end	EPSILON	0.241523	4	4085	3168.66	0.7563	0.7393
coverage_end	VARIANT × EPSILON	0.004947	8	4085	32.45	0.0598	0.0147

Table S5a reports a standard two-way ANOVA decomposition (main effects + interaction, df_interaction = 8), whereas Supplementary Table S5 and Core Table 3 report the full cell-means model (df = 14, combining main effects and interaction). F-values differ accordingly; η^2_p for the cell-means model is reported in Core Table 3.

Supplementary Table S5b. Kruskal-Wallis

Metric	Test	Condition	H	p
**Selectivity_end**	VARIANT	ε=0	84.82	3.82e-19
**Selectivity_end**	VARIANT	ε=1	67.54	2.16e-15
**Selectivity_end**	VARIANT	ε=2	58.44	2.04e-13
**Selectivity_end**	VARIANT	ε=3	65.77	5.22e-15
**Selectivity_end**	VARIANT	ε=5	19.13	7.02e-05
**Selectivity_end**	EPSILON	original	7.82	9.84e-02
**Selectivity_end**	EPSILON	rewired_type	4.69	3.20e-01
**Selectivity_end**	EPSILON	lesion_KC_MBON	48.45	7.60e-10
**coverage_end**	VARIANT	ε=0	311.51	2.27e-68
**coverage_end**	VARIANT	ε=1	31.1	1.77e-07
**coverage_end**	VARIANT	ε=2	15.55	4.21e-04
**coverage_end**	VARIANT	ε=3	19.67	5.35e-05
**coverage_end**	VARIANT	ε=5	12.81	1.65e-03
**coverage_end**	EPSILON	original	78.02	4.57e-16
**coverage_end**	EPSILON	rewired_type	1365.7	1.89e-294
**coverage_end**	EPSILON	lesion_KC_MBON	1514.28	0.00e+00

Supplementary Results

S6. Disorder sensitivity in connectome benchmarks (summary figures)

These figures summarize disorder sensitivity in the connectome benchmarks discussed in the main text. The key point is that disorder-enhanced selectivity is circuit-dependent: Drosophila exhibits a pronounced non-monotonic peak at intermediate ε in the native topology, whereas the C. elegans touch circuit shows a monotonic increase over the scanned range. Supplementary Figure S2 retains the full cross-scale illustration, with the mouse cortex proxy shown explicitly as exploratory supplementary context rather than as main-text evidence.

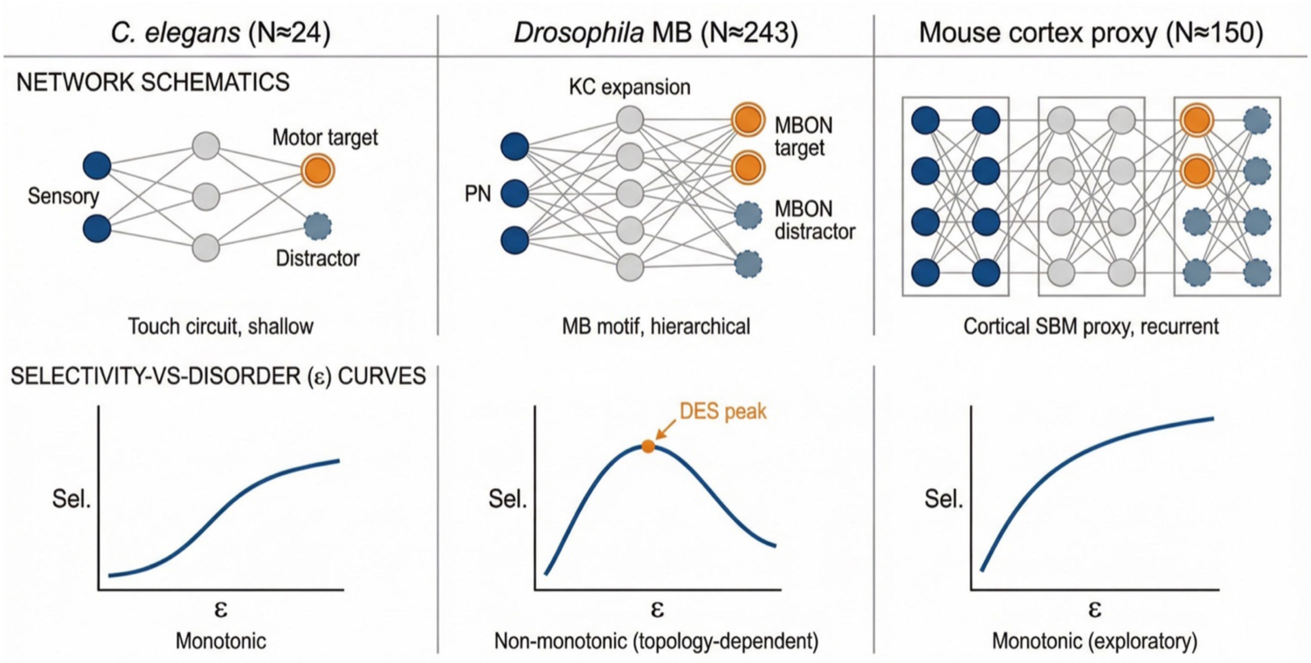

Supplementary Figure S1. Universal hierarchical decision architecture schematic across scales (nematode → insect → mammal) with representative disorder-sensitivity profiles (schematic only). Quantitative curves are shown in Supplementary Figure S2.

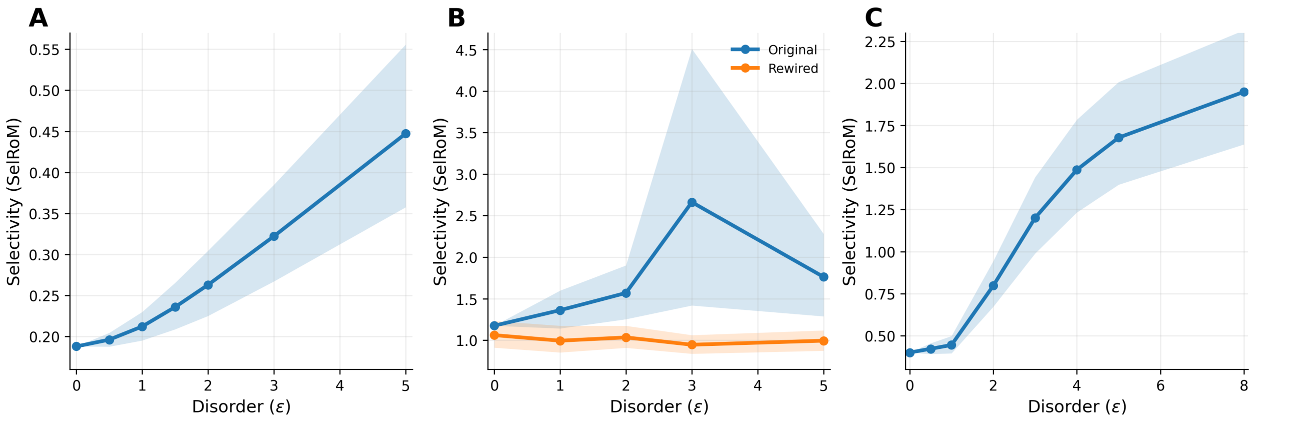

Supplementary Figure S2. Disorder dependence of selectivity across connectome benchmarks. Selectivity ratio-of-means (SelRoM) versus disorder ε at κ=0.001 for (A) C. elegans touch circuit, (B) Drosophila larva mushroom-body motif (original vs. degree-preserving rewired), and (C) the exploratory mouse cortex proxy (hierarchical SBM). Lines show means and shaded bands show 95% bootstrap confidence intervals across trial or replicate ensembles. Core Figure 2 reproduces only the biological panels (A-B).

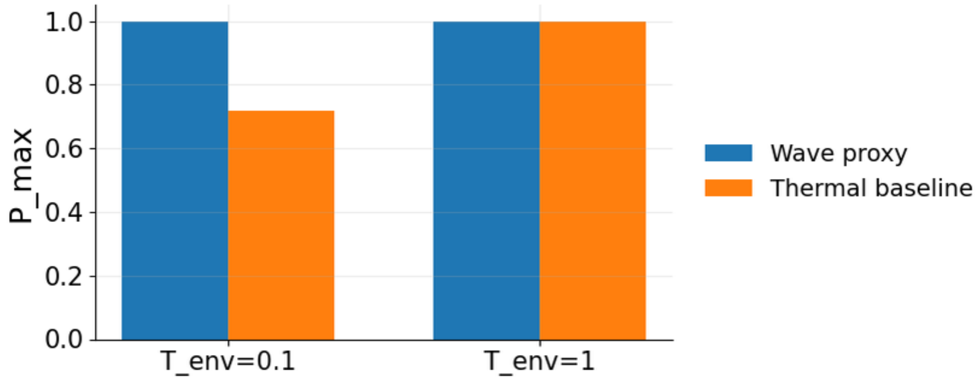

Supplementary Figure S3. C. elegans touch circuit: maximum target absorption P_max at T_env=1.0 and T_env=0.1 for the wave proxy vs. thermal baseline.

S7. *Drosophila* larva connectome benchmark

Supplementary Tables S3a-S3b. Compact summary (Drosophila benchmark). Summarizes best-by-objective GKSL performance as a function of disorder ε, alongside CRW and thermal baselines. Note: for ε=5, the best-by-selectivity metric is undefined under the minimum-throughput filter used in the sweep.

Supplementary Table S3a. GKSL summary (Drosophila benchmark).

ε	κ_sel	Sel	cov	P_T	P_D	κ_IPC	IPC
**0**	1.0	1.520	0.0122	0.0074	0.0048	1.0	0.1134
**1**	1.0	1.558	0.0094	0.0057	0.0037	1.0	0.1058
**2**	0.3	1.544	0.0084	0.0051	0.0033	1.0	0.1064
**3**	0.001	2.224	0.0079	0.0055	0.0025	0.001	0.1676
**5**	—	—	—	—	—	1.0	0.0607

Supplementary Table S3b. Classical baselines (CRW and thermal).

ε	Sel_CRW	IPC_CRW	Sel_T0.1	IPC_T0.1	Sel_T1.0	IPC_T1.0
**0**	0.878	-0.0635	0.878	-0.0635	0.878	-0.0635
**1**	0.878	-0.0635	0.907	-0.0469	0.975	-0.0123
**2**	0.878	-0.0635	1.274	0.1157	0.937	-0.0319
**3**	0.878	-0.0635	1.252	0.1066	1.037	0.0176
**5**	0.878	-0.0635	1.167	0.0736	0.983	-0.0082

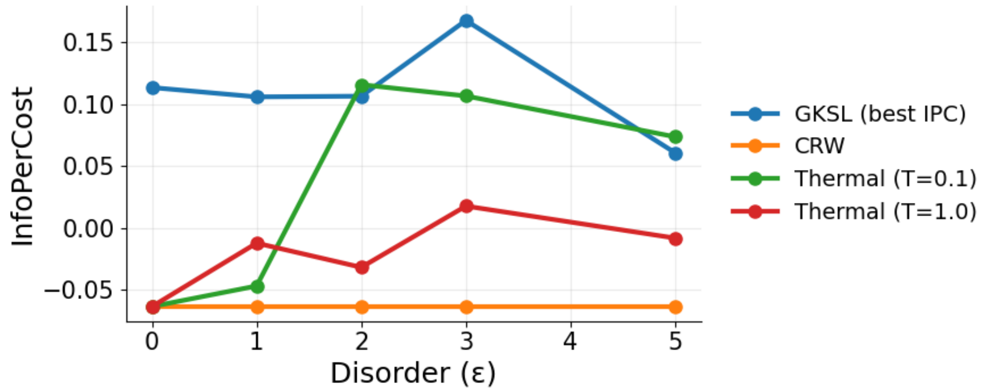

Supplementary Figure S4. Best-by-InfoPerCost across disorder ε.

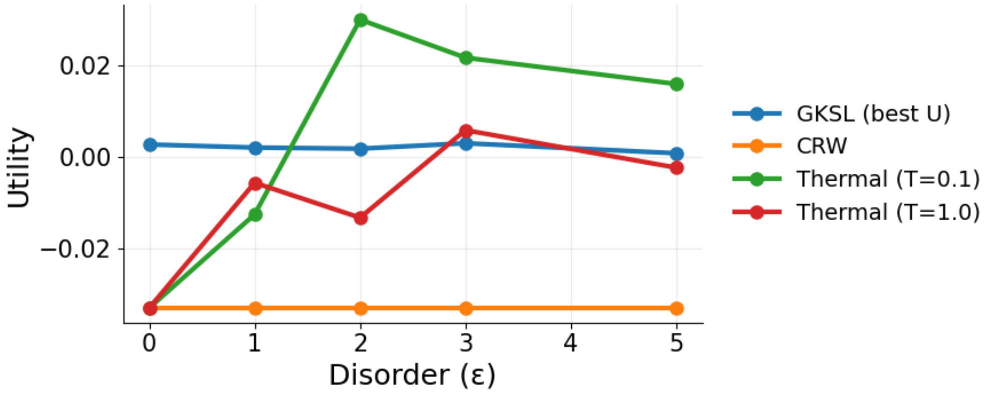

Supplementary Figure S5. Best-by-Utility across disorder ε.

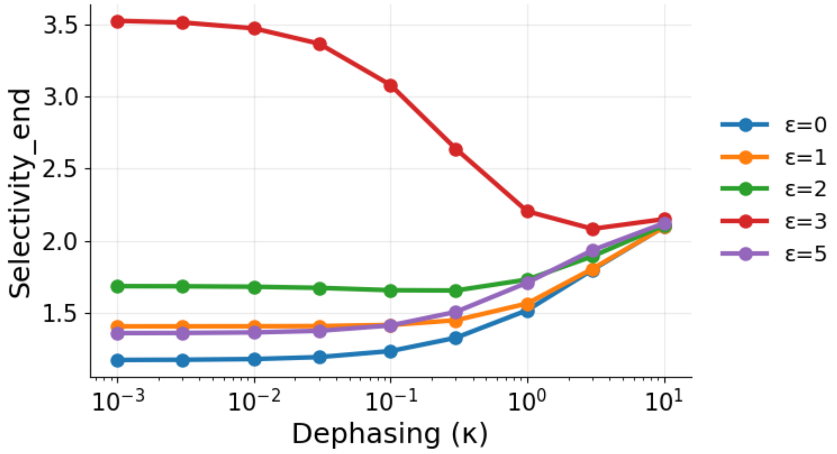

Supplementary Figure S6. Selectivity_end vs. κ for multiple ε. Supporting/diagnostic analysis; not required for the main conclusions.

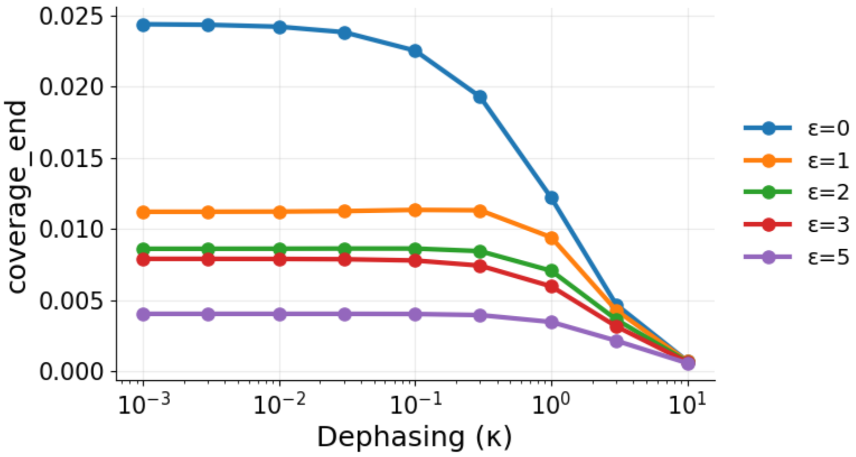

Supplementary Figure S7. coverage_end vs. κ for multiple ε. Supporting/diagnostic analysis; not required for the main conclusions.

S8. Localization diagnostics

We report participation-ratio diagnostics and differential localization
$\Delta PR = PR_{target} - PR_{distractor}$

Supplementary Table S4. Localization diagnostics by ε (κ=0.001). At ε=0 (no disorder), energies are fixed, and dynamics are deterministic, hence the across-trial std=0.

ε	n_{trials}	Selectivity_end_mean	Selectivity_end_std	$delta_{PR}$_mean	$delta_{PR}$_std	$ratio_{PR}$_mean	$ratio_{PR}$_std
**0**	10	1.175	0	-1.961	2.341e-16	0.6072	0
**1**	10	1.408	0.3943	-0.7682	1.175	0.7978	0.3145
**2**	10	1.687	0.8646	-0.1843	0.5956	0.9599	0.1628
**3**	10	3.524	4.945	-0.09162	1.232	1.054	0.4415
**5**	10	1.361	1.249	0.05169	1.045	1.082	0.4161

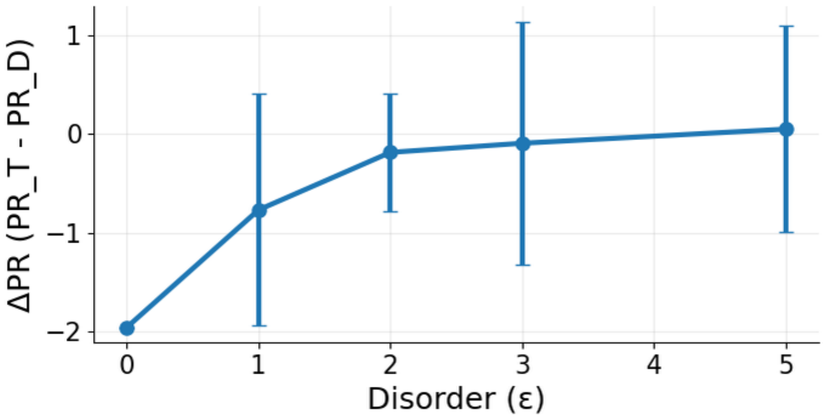

Supplementary Figure S8. ΔPR vs ε at κ=0.001.

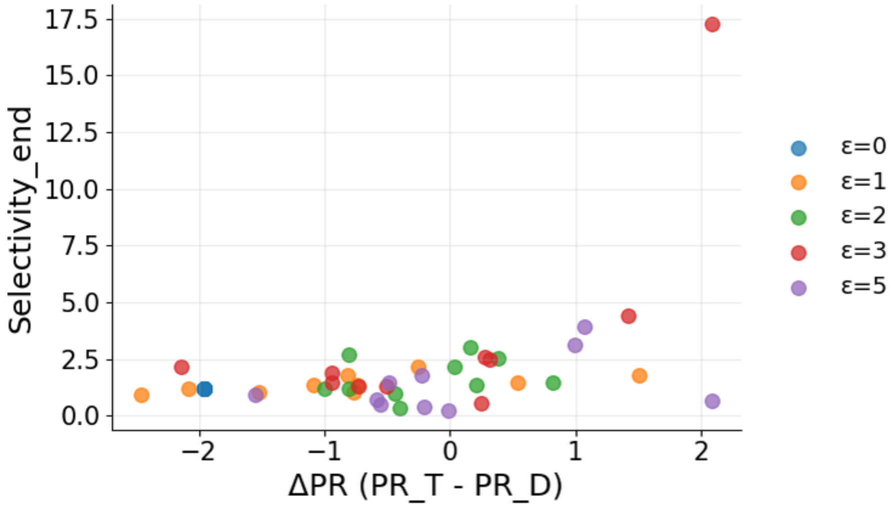

Supplementary Figure S9. Selectivity_end vs ΔPR scatter.

S9. Architecture dependence and negative controls

Two-way ANOVA and bootstrap tests quantify architecture × disorder effects and the dependence of the DES peak on topology.

Supplementary Table S5. Two-way ANOVA (κ=0.001).

metric	effect	F	df1	df2	pvalue
**Selectivity_end**	VARIANT	44.7	2	4097	6.25e-20
**Selectivity_end**	EPSILON	31.12	4	4095	1.45e-25
**Selectivity_end**	VARIANT×EPSILON*	20.53	14	4085	4.16e-51
**coverage_end**	VARIANT	14.33	2	4097	6.28e-07
**coverage_end**	EPSILON	2907	4	4095	<1e-300
**coverage_end**	VARIANT×EPSILON*	932.4	14	4085	<1e-300

* VARIANT×EPSILON denotes the full cell-means model (including interaction terms).

Supplementary Table S6. Bootstrap DES within variants (κ=0.001).

variant	contrast	delta	CI_{low}	CI_{high}	significance
**lesion_KC_MBON**	DES = Selectivity_end(ε=3) - Selectivity_end(ε=0)	1.102	0.83	1.398	significant
**lesion_KC_MBON**	Selectivity_end(ε=3) - Selectivity_end(ε=5)	-0.502	-0.832	-0.188	significant
**original**	DES = Selectivity_end(ε=3) - Selectivity_end(ε=0)	1.489	0.249	3.355	significant
**original**	Selectivity_end(ε=3) - Selectivity_end(ε=5)	-0.898	-2.805	0.491	ns
**rewired_type**	DES = Selectivity_end(ε=3) - Selectivity_end(ε=0)	0.162	0.056	0.274	significant
**rewired_type**	Selectivity_end(ε=3) - Selectivity_end(ε=5)	0.436	0.21	0.672	significant

Supplementary Table S7. Bootstrap DES between variants (κ=0.001).

contrast	delta	CI_{low}	CI_{high}	significance
**DES(original) - DES(rewired_type)**	1.328	0.069	3.232	p<0.001
**DES(original) - DES(lesion_KC_MBON)**	0.383	-0.902	2.261	ns
**DES(lesion_KC_MBON) - DES(rewired_type)**	0.938	0.646	1.254	p<0.001

Supplementary Table S8. Architecture dependence metrics (κ=0.001). Note: the original (native) graph has nruns_total=20 because there is only one fixed connectome instance; lesion and rewired variants use 20 surrogates × 20 disorder/energy seeds (400 runs) to estimate variability.

variant	ε	nrunsTotal	meanPTend	meanPDend	SelEndMean	SelRoM	covEndMean
**lesion_KC_MBON**	0	400	0.01647	0.01396	1.194	1.18	0.03042
**lesion_KC_MBON**	1	400	0.009295	0.007022	1.454	1.324	0.01632
**lesion_KC_MBON**	2	400	0.007125	0.004964	1.702	1.435	0.01209
**lesion_KC_MBON**	3	400	0.006401	0.003987	2.296	1.605	0.01039
**lesion_KC_MBON**	5	400	0.002816	0.002368	1.795	1.189	0.005184
**original**	0	20	0.01319	0.01122	1.175	1.175	0.02441
**original**	1	20	0.006436	0.005012	1.362	1.284	0.01145
**original**	2	20	0.00472	0.003596	1.569	1.312	0.008316
**original**	3	20	0.004564	0.002589	2.662	1.763	0.007153
**original**	5	20	0.002089	0.00156	1.762	1.339	0.003649
**rewired_type**	0	400	0.0126	0.01254	1.062	1.005	0.02515
**rewired_type**	1	400	0.008359	0.008874	1.113	0.9419	0.01723
**rewired_type**	2	400	0.006022	0.006077	1.255	0.991	0.0121
**rewired_type**	3	400	0.004412	0.004895	1.225	0.9014	0.009307
**rewired_type**	5	400	0.002553	0.002638	1.659	0.9681	0.005191

Supplementary Table S8a. Consolidated permutation summary for the Drosophila DES contrast Δ(ε=3−ε=0). The first row reports the primary A8 architecture benchmark at κ=0.001 (n=20); the remaining rows report the companion A7 κ-sweep robustness branch in the native Drosophila topology (n=10 per κ).

Branch	κ	Δ(ε=3−ε=0)	p_perm	n	Interpretation
**A8 primary architecture**	0.001	1.487	0.010	20	primary confirmatory
**A7 companion κ-sweep**	0.001	2.349	0.008	10	low-κ robust
**A7 companion κ-sweep**	0.01	2.291	0.008	10	low-κ robust
**A7 companion κ-sweep**	0.1	1.843	0.006	10	detectable
**A7 companion κ-sweep**	1.0	0.685	0.011	10	weakened but detectable
**A7 companion κ-sweep**	3.0	0.284	0.047	10	borderline
**A7 companion κ-sweep**	10.0	0.053	0.203	10	high-κ non-significant

Supplementary Table S8b. Gamma log-link GLM sensitivity analysis (Drosophila A8 architecture benchmark, $\kappa = 0.001$).

Model	Contrast	Ratio	95% CI	p_Wald	n
**M1 naive**	ε=3 vs ε=0	2.265	1.422 – 3.607	5.7×10^{-4}	100
**M2 conservative**	ε=3 vs ε=1	1.955	1.162 – 3.288	0.012	80
**M3 topological**	native×ε=3 interaction	1.963	1.178 – 3.273	0.010	4100

M1 fits Selectivity_end ~ C(ε), Gamma(log), on the 100 native-architecture rows (20 per ε level). Because the ε = 0 cell is deterministic on a single native graph (all 20 rows collapse to one unique value), M2 drops ε = 0 and uses ε = 1 as the reference level; this is the conservative primary sensitivity estimate. M3 fits Selectivity_end ~ C(variant) × C(ε), Gamma(log), on all 4,100 benchmark rows with the degree-preserving rewired surrogate as the reference variant; the reported ratio-of-ratios isolates the topological component of the DES contrast. All three models converged. This analysis is a sensitivity complement to the permutation-based primary test (Table S8a) and does not replace it.

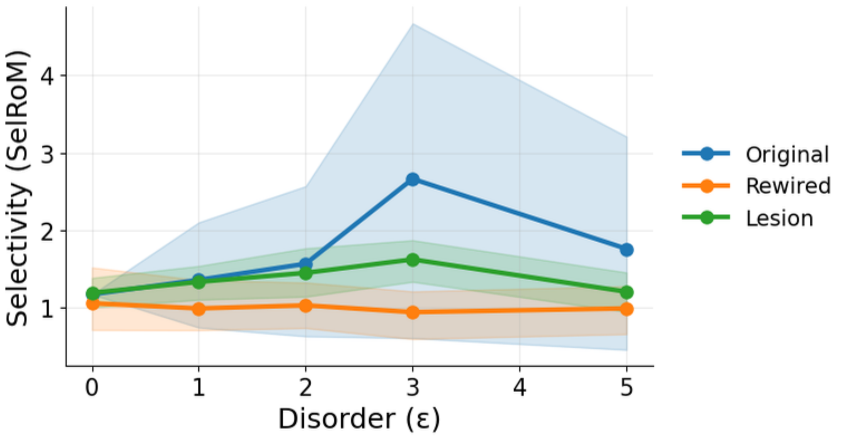

Supplementary Figure S10. Selectivity ratio-of-means vs ε at κ=0.001.

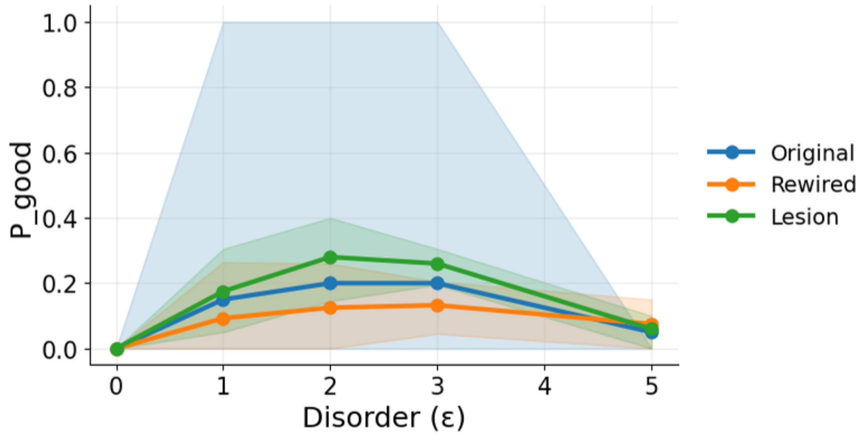

Supplementary Figure S11. P_good vs ε at κ=0.001 (S_thr=2.0, pT_min=0.005).

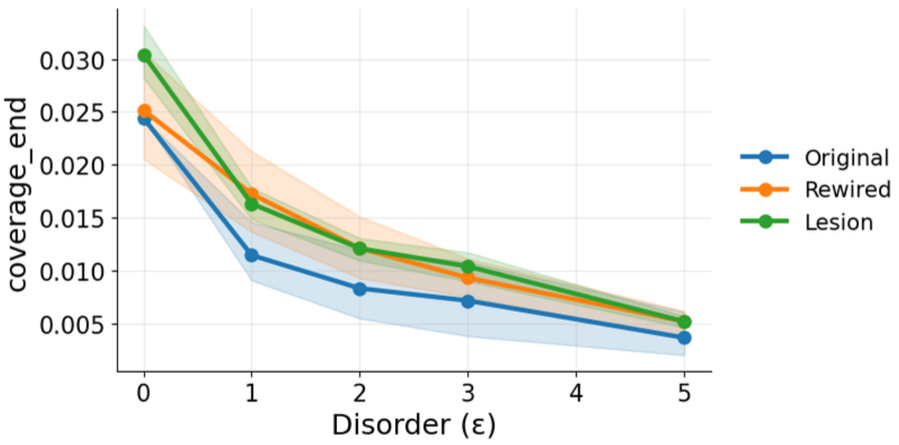

Supplementary Figure S12. coverage_end vs ε at κ=0.001.

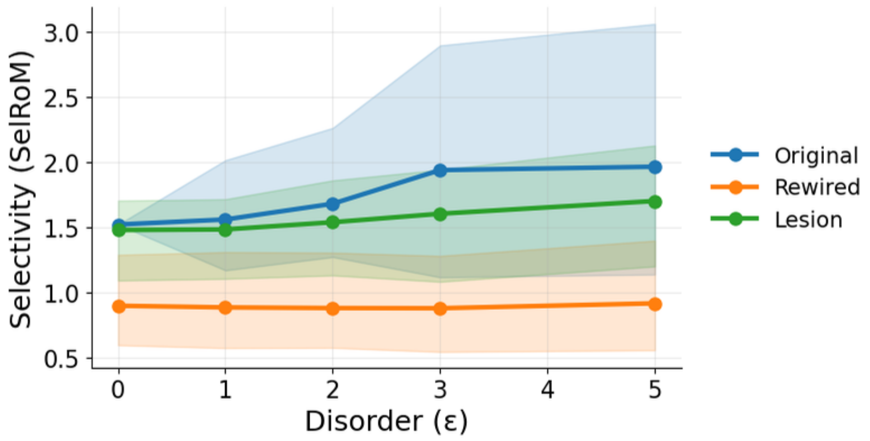

Supplementary Figure S13. Selectivity ratio-of-means vs ε at κ=1.0.

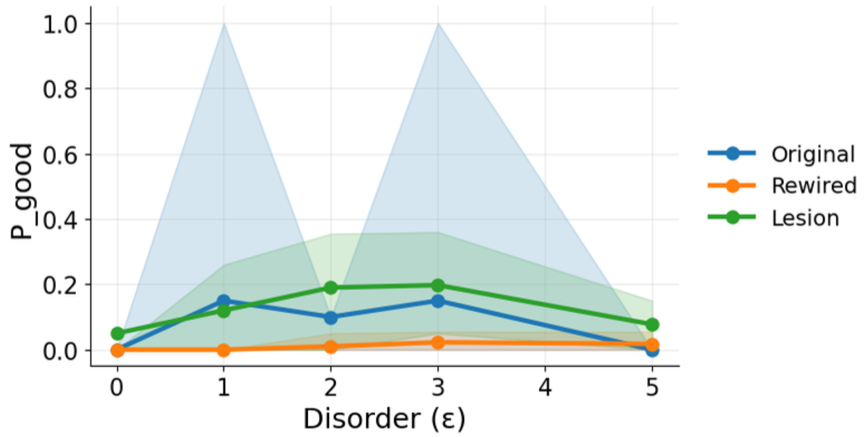

Supplementary Figure S14. P_good vs ε at κ=1.0 (S_thr=2.0, pT_min=0.005).

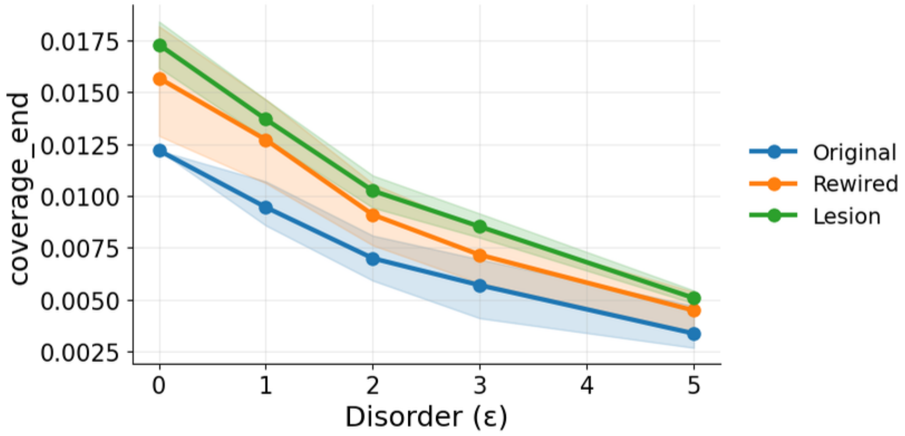

Supplementary Figure S15. coverage_end vs ε at κ=1.0.

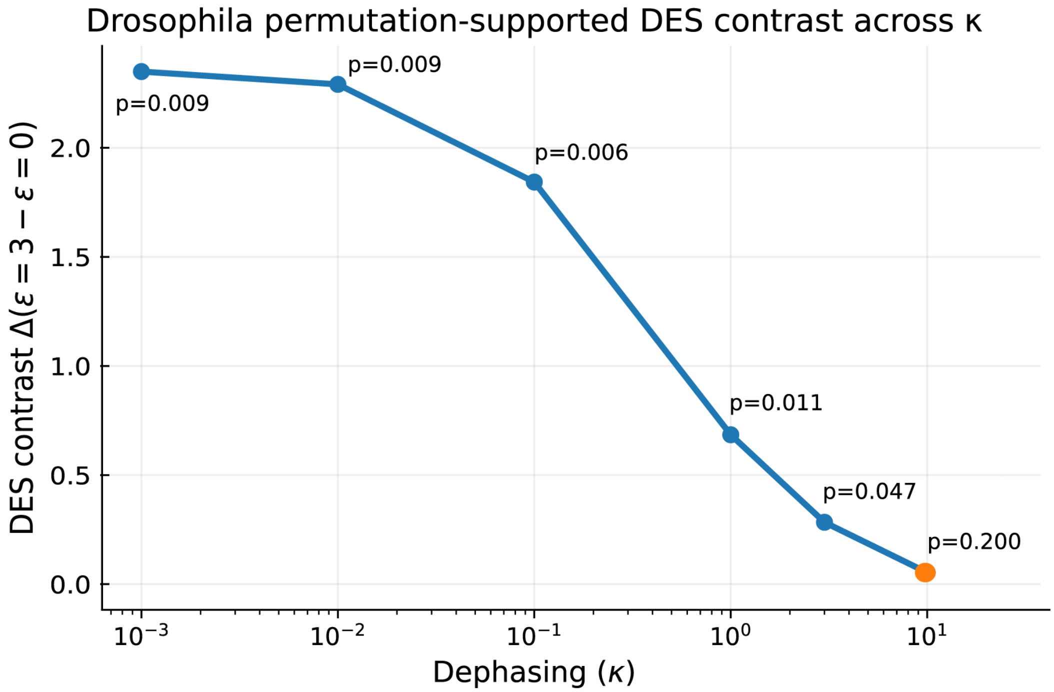

Supplementary Figure S15a. Companion κ-sweep benchmark for the native Drosophila topology: permutation-based DES contrast Δ(ε=3−ε=0) across κ values. This robustness branch is separate from the primary κ=0.001 architecture benchmark. The contrast is strongest at low κ, weakens progressively with increasing dephasing, and becomes non-significant in the high-κ regime at κ=10.0.

S10. Exploratory mouse cortical proxy: energy–selectivity trade-off

Summary tables from the exploratory mouse cortical proxy analyses are reproduced below. Supplementary Table S10a reports the disorder (ε) sweep at fixed T_env=0.1 corresponding to Supplementary Figure S2C.

Supplementary Table S9. Mouse cortex proxy: transport summary (T_env sweep).

T_env	n	recallMean	recallCI95	leakageMean	leakageCI95	SelRoM
**0.1**	100	0.214	±0.021	0.041	±0.009	5.22
**0.3**	100	0.287	±0.026	0.052	±0.010	5.52
**1.0**	100	0.198	±0.019	0.067	±0.011	2.96
**3.0**	100	0.112	±0.014	0.083	±0.013	1.35

Supplementary Table S10. Mouse cortex proxy: memory summary (κ sweep).

T_env	n	recallFracMean	recallMinusLeakageMean	recallMinusLeakageCI95
**0.1**	100	0.84	0.173	±0.018
**0.3**	100	0.85	0.235	±0.021
**1.0**	100	0.75	0.131	±0.017
**3.0**	100	0.57	0.029	±0.012

Supplementary Table S10a. Cortex proxy disorder (ε) sweep at T_env=0.1 (n_trials=100; seed=1; T_end=100). SelRoM denotes selectivity as ratio-of-means Recall/Leakage; SelRoM_CI95 is a 95% bootstrap confidence interval over trials.

ε	n	recallMean	recallCI95	leakageMean	leakageCI95	SelRoM	SelRoM_CI95
**0**	100	0.267	0.000	0.667	0.000	0.40	[0.40, 0.40]
**1**	100	0.288	0.023	0.647	0.024	0.44	[0.40, 0.50]
**2**	100	0.402	0.040	0.504	0.040	0.80	[0.67, 0.94]
**3**	100	0.480	0.046	0.400	0.042	1.20	[0.99, 1.44]
**4**	100	0.517	0.048	0.348	0.039	1.49	[1.23, 1.78]
**5**	100	0.536	0.048	0.319	0.037	1.68	[1.40, 2.01]
**8**	100	0.558	0.047	0.286	0.034	1.95	[1.64, 2.32]

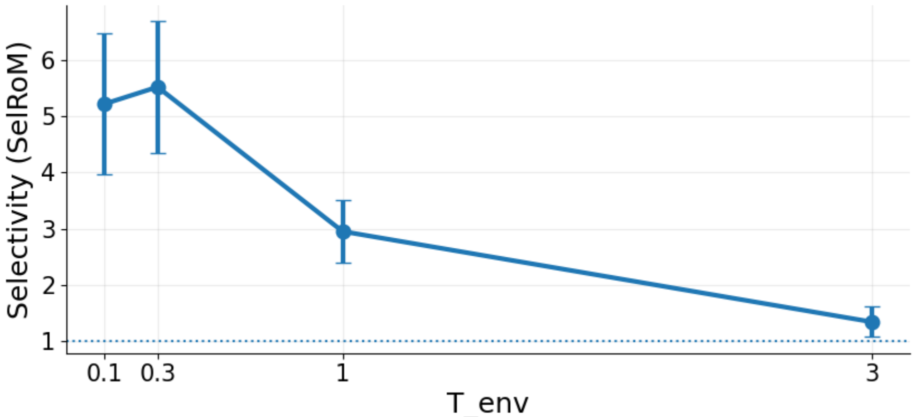

Supplementary Figure S16. Mouse cortex proxy: selectivity SelRoM versus temperature parameter T_env. Error bars show an approximate 95% CI (delta-method propagation from recall/leakage CIs in Table S9); the horizontal line at SelRoM=1 indicates parity.

S11. Exploratory evolutionary game-theoretic selection for modular regime separation

This section provides an auxiliary exploratory plausibility argument. Motivated by the permeability paradox discussed in the Core, the analysis asks whether modular separation of regimes (a transport-optimized module versus a memory-optimized module) can be favored by selection under mixed task demands. It is not used as primary evidence for the central Stage-I claim.

Setup. We consider two objective classes: (i) fast transport/selection on a disordered substrate, which in the cortex-proxy transport benchmark is optimized at intermediate dephasing ($\kappa \approx 0.5$), and (ii) memory/retention, which is optimized at much lower dephasing ($\kappa \approx 0.01$) to minimize leakage and preserve attractor stability. Strategies compete either as global controls (single κ for all modules) or as modular controls (distinct κ_transport and κ_memory). This trade-off is the modeling counterpart of the permeability paradox: routing benefits from permeability, whereas storage benefits from confinement.

Fitness is defined as a weighted mixture of the two objectives (p_transport for transport and 1−p_transport for memory) minus a modularity cost p_modularity. We sweep 90 strategies across a 21×41 environment grid (861 environments) spanning p_transport and p_modularity, and evaluate which strategy maximizes fitness in each environment.

Result. Modular specialization is favored across mixed environments (roughly p_transport≈0.2–0.8), provided modularity costs remain below ≈0.16 (strict robustness consensus). In this modular-winning region, modular strategies show median fitness gains of ≈12% over the best global strategy, with typical gains ≈7%–31% (maximum ≈31%) (Supplementary Figures S17–S18).

Under standard replicator dynamics with illustrative parameters (p_transport=0.5, p_modularity=0.05), the modular strategy can exceed 99% population share within about 15 generations in an illustrative run, consistent with the fitness advantage shown in the phase diagram (Figures S17–S18).

Interpretation and caveats. This analysis is stylized and does not constitute empirical evidence for a specific biological substrate. Its purpose is architectural only: it illustrates why local tuning of coherence/noise regimes (transport vs. memory) could be evolutionarily stable when cognitive systems face competing objectives, but it should be read as supplementary plausibility context rather than direct comparative evidence.

Availability. The complete analysis bundle and public numeric backends are archived at Zenodo (DOI: 10.5281/zenodo.18379850) and are also included in the reproducibility repository under experiments/step6_releasecandidate.

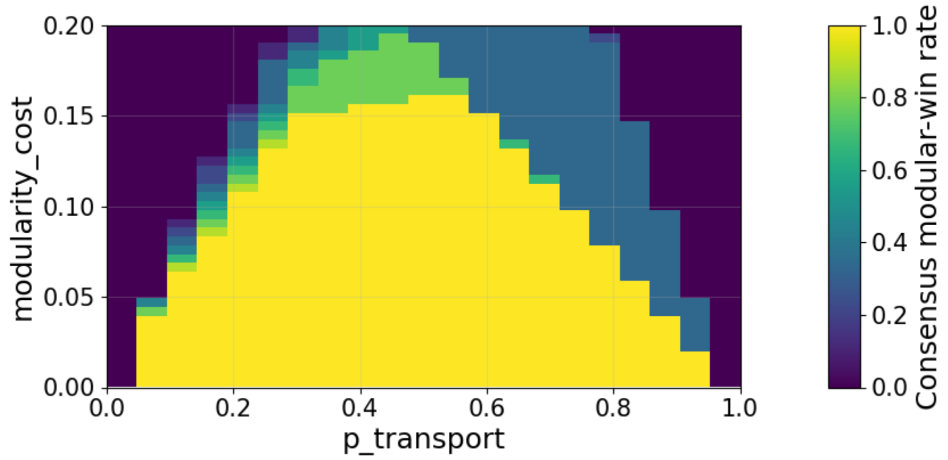

Supplementary Figure S17. Robustness audit for the evolutionary-game phase diagram. Consensus modular-win rate (0–1) across metric variants over the environment grid (p_transport × modularity_cost).

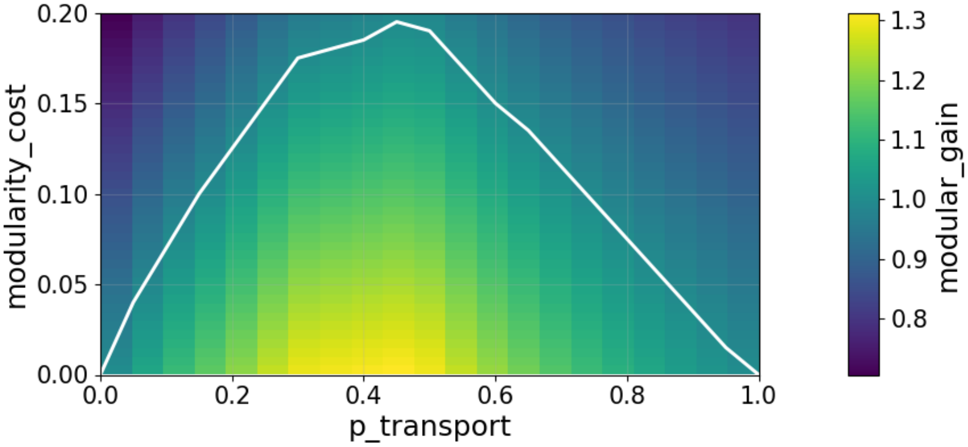

Supplementary Figure S18. Evolutionary game-theoretic phase diagram for modular specialization. Region where modular specialization is favored as a function of the transport–memory environment mix (p_transport) and the modularity cost (p_modularity). Supporting/diagnostic analysis; not required for the main conclusions.

Supplementary References

Anderson, P.W. (1958). Absence of diffusion in certain random lattices. Phys Rev. 109(5):1492-1505. doi:10.1103/PhysRev.109.1492.

Anokhin, K.V. (2021). The Cognitome: Seeking the Fundamental Neuroscience of a Theory of Consciousness. Neurosci Behav Physiol. 51:915-937. doi:10.1007/s11055-021-01149-4.

Attwell, D., and Laughlin, S.B. (2001). An energy budget for signaling in the grey matter of the brain. J Cereb Blood Flow Metab. 21(10):1133-1145. doi:10.1097/00004647-200110000-00001.

Caruso, F., Chin, A.W., Datta, A., Huelga, S.F., and Plenio, M.B. (2009). Highly efficient energy excitation transfer in light-harvesting complexes: the fundamental role of noise-assisted transport. J Chem Phys. 131:105106. doi:10.1063/1.3213548.

Dolgikh, O. (2026). Evolutionary Game Theory Reveals Modular Specialization as a Universal Solution to Neural Trade-offs (companion dataset). Zenodo. doi:10.5281/zenodo.18379850.

Dolgikh, O. (2026). CRN Drosophila Larva Mushroom-Body Benchmark: Simulation Artifacts and Derived Data. Zenodo. doi:10.5281/zenodo.18697116.

Engel, G.S., Calhoun, T.R., Read, E.L., Ahn, T.-K., Mancal, T., Cheng, Y.-C., et al. (2007). Evidence for wavelike energy transfer through quantum coherence in photosynthetic systems. Nature. 446:782-786. doi:10.1038/nature05678.

Friston, K.J. (2010). The free-energy principle: a unified brain theory? Nat Rev Neurosci. 11(2):127-138. doi:10.1038/nrn2787.

Gammaitoni, L., Hänggi, P., Jung, P., and Marchesoni, F. (1998). Stochastic resonance. Rev Mod Phys. 70(1):223-287. doi:10.1103/RevModPhys.70.223.

Gorini, V., Kossakowski, A., and Sudarshan, E.C.G. (1976). Completely positive dynamical semigroups of N-level systems. J Math Phys. 17(5):821-825. doi:10.1063/1.522979.

Hopfield, J.J. (1982). Neural networks and physical systems with emergent collective computational abilities. Proc Natl Acad Sci U S A. 79(8):2554-2558. doi:10.1073/pnas.79.8.2554.

Howarth, C., Gleeson, P., and Attwell, D. (2012). Updated energy budgets for neural computation in the neocortex and cerebellum. J Cereb Blood Flow Metab. 32(7):1222-1232. doi:10.1038/jcbfm.2012.35.

Kendon, V. (2007). Decoherence in quantum walks - a review. Math Struct Comput Sci. 17(6):1169-1220. doi:10.1017/S0960129507006354.

Landauer, R. (1961). Irreversibility and heat generation in the computing process. IBM J Res Dev. 5(3):183-191. doi:10.1147/rd.53.0183.
Lennie, P. (2003). The cost of cortical computation. Curr Biol. 13(6):493-497. doi:10.1016/S0960-9822(03)00135-0.
Levy, W.B., and Calvert, V.G. (2021). Communication consumes 35 times more energy than computation in the human cortex, but both costs are needed to predict synapse number. Proc Natl Acad Sci U S A. 118(18):e2008173118. doi:10.1073/pnas.2008173118.
Lindblad, G. (1976). On the generators of quantum dynamical semigroups. Commun Math Phys. 48:119-130. doi:10.1007/BF01608499.
Maslov, S., and Sneppen, K. (2002). Specificity and stability in topology of protein networks. Science. 296(5569):910-913. doi:10.1126/science.1065103.
Oh, S.W., Harris, J.A., Ng, L., Winslow, B., Cain, N., Mihalas, S., et al. (2014). A mesoscale connectome of the mouse brain. Nature. 508:207-214. doi:10.1038/nature13186.
Padamsey, Z., Katsanevaki, D., Dupuy, N., and Rochefort, N.L. (2022). Neocortex saves energy by reducing coding precision during food scarcity. Neuron. 110:280-296.e10. doi:10.1016/j.neuron.2021.10.024.
Plenio, M.B., and Huelga, S.F. (2008). Dephasing-assisted transport: quantum networks and biomolecules. New J Phys. 10:113019. doi:10.1088/1367-2630/10/11/113019.
Rao, R.P.N., and Ballard, D.H. (1999). Predictive coding in the visual cortex: a functional interpretation of some extra-classical receptive-field effects. Nat Neurosci. 2:79-87. doi:10.1038/4580.
Rebentrost, P., Mohseni, M., and Aspuru-Guzik, A. (2009). Role of quantum coherence and environmental fluctuations in chromophoric energy transport. J Phys Chem B. 113(29):9942-9947. doi:10.1021/jp901159h.
Varshney, L.R., Chen, B.L., Paniagua, E., Hall, D.H., and Chklovskii, D.B. (2011). Structural properties of the Caenorhabditis elegans neuronal network. PLoS Comput Biol. 7(2):e1001066. doi:10.1371/journal.pcbi.1001066.
Whitfield, J.D., Rodríguez-Rosario, C.A., and Aspuru-Guzik, A. (2010). Quantum stochastic walks: a generalization of classical random walks and quantum walks. Phys Rev A. 81:022323. doi:10.1103/PhysRevA.81.022323.
Winding, M., Pedigo, B.D., Barnes, C.L., Patsolic, H.G., Park, Y., Kazimiers, T., et al. (2023). The connectome of an insect brain. Science. 379(6636):eadd9330. doi:10.1126/science.add9330.
Yerkes, R.M., and Dodson, J.D. (1908). The relation of strength of stimulus to rapidity of habit-formation. J Comp Neurol Psychol. 18:459-482.
